# Menstrual health: an analysis of experiences among school-aged adolescents

**DOI:** 10.1590/1980-220X-REEUSP-2025-0003en

**Published:** 2026-01-19

**Authors:** Jennifer Caroline de Oliveira Adomaitis, Natália Sevilha Stofel, Maria Gabriele Formis Silva, Vivian Campos Santos, Flávio Adriano Borges, Diene Monique Carlos, Bruna Luana de Souza Farias, Márcia Niituma Ogata, Juliana Cristina dos Santos Monteiro

**Affiliations:** 1 Universidade Federal de São Carlos, Departamento de Enfermagem, São Carlos, SP, Brazil.; 2 Universidade de São Paulo, Escola de Enfermagem de Ribeirão Preto, Ribeirão Preto, SP, Brazil.; 3 Universidade Federal de São Carlos, Programa de Pós-Graduação em Enfermagem, São Carlos, SP, Brazil.

**Keywords:** Adolescent, Menstruation, School

## Abstract

**Objective:**

To understand the perceptions of school adolescents about the menstrual cycle.

**Method:**

This was an exploratory mixed-methods study conducted at a State Technical School in the state of São Paulo. Descriptive statistical analysis was performed for the quantitative data, and Iramuteq software was used for the qualitative data. The findings were compared with the principles of Reproductive Justice.

**Results:**

Thirty-four adolescents participated in this study. It was found that 17% had difficulty purchasing sanitary pads, 88% felt insecure when menstruating, and 53% had missed school because they were menstruating. All stated that they had learned about menstruation at school. Five thematic categories emerged: 1) Challenges of Menstruation at School; 2) Education and Conversations about Menstruation in the Family Environment; 3) Support and Counseling; 4) Physical and Practical Difficulties of Menstruation; 5) Shame and Stigmatization.

**Conclusion:**

The research reveals that menstruation significantly impacts the lives of adolescents, causing insecurity and absenteeism. Schools play a significant role in disseminating information on the subject, and the data obtained point to the need to broaden the debate and reduce stigma.

## INTRODUCTION

Although menstruation is a physiological process, it is often accompanied not only by physical discomfort and emotional changes, but also by stigma, and is considered taboo. In many cultures, there is a tendency to associate menstruation with something impure or negative^(1)^. Patriarchy, as a normalizer of bodies and social behaviors, when focusing on the menstrual cycle, produces and reproduces misinformation, creating pejorative views and placing menstruation in a position that arouses disgust^(2)^. Having this view associated with the biologically female body further segregates people who menstruate^(3)^.

Menstrual poverty refers to the difficulty or lack of access to essential management products during the menstrual period, as well as the unavailability of adequate places to use them. This includes the absence of basic sanitation services and limited access to information about menstruation^(4)^. In essence, menstrual poverty is a serious social problem that reflects social and gender inequalities, especially when we compare the high cost of menstrual management products with other personal care items^(5)^. Menstruation is invisible, meaning it is not discussed, and the problems are not observed, but they do not disappear and have a profound impact on the most vulnerable people^(6)^.

In addition to the difficulty of accessing products and the lack of infrastructure, it is important to consider that menstruation involves a series of needs related to physical, emotional, and social health. The concept of menstrual health is defined as a state of physical, mental, and social well-being in relation to the menstrual cycle, and not just the absence of disease or illness. This approach broadens the view beyond material conditions to also include the right to information about menarche, access to safe and private spaces for menstrual hygiene, and the possibility of full participation in educational, social, and professional activities during the menstrual period^(7)^.

Recognizing menstrual health as a fundamental right implies considering that both menstrual poverty and the lack of adequate menstrual health care contribute to negative impacts on the lives of people who menstruate. Among the impacts generated, one of the most significant is school absenteeism. According to data from a United Nations Population Fund (UNPF) study, one in 10 girls miss school when they are menstruating^(8)^. In Brazil, according to data from the Menstrual Dignity Program implementation guide, one in four girls misses school when they are menstruating because they cannot afford sanitary pads; about 4 million girls suffer from at least one deprivation of resources in schools (access to sanitary pads and/or basic facilities, such as bathrooms and soap), and only 20% of students felt well informed when they experienced their first menstruation^(9)^.

The lack of information and access to menstrual management products, combined with prejudice and difficulties, causes discomfort, embarrassment, and even bullying, resulting in the exclusion of girls from various daily activities and negatively impacting their academic and professional performance, compared to people who do not menstruate^(10)^. In addition, stigma causes girls to avoid changing their sanitary pads at school, which compromises their physical health. “Menstrual etiquette” also reinforces the idea that one should not talk openly about menstruation, which undermines girls’ self-esteem and confidence. This stigma is based on the belief that menstrual blood is dirty or impure, something to be avoided^(11)^.

Other factors that contribute to absenteeism are the physical symptoms associated with menstruation, such as dysmenorrhea, which is characterized by intense menstrual cramps of uterine origin. One study indicates that this condition affects up to 95% of menstruating women^(12)^; Premenstrual Syndrome, characterized by a set of physical, emotional, and behavioral manifestations that arise during the luteal phase of the menstrual cycle (usually in the week before menstruation) and tend to disappear with the onset of menstrual bleeding^(13)^.

In addition to physical symptoms, menstruation also has significant emotional impacts. A cross-sectional study conducted in Japan in 2022 with 198 high school students revealed that menstrual symptoms, such as severe pain (dysmenorrhea), impact on daily activities, unexpected menstruation, and frequent need for pain medication, were strongly associated with lower quality of life scores, especially in aspects related to mental health^(14)^. The impacts of these symptoms are profound and can compromise quality of life, interfering with daily activities and leading to school, professional, and social exclusion. Despite its high prevalence, many people face insufficient or inhumane health support^(15)^.

The reality that around 800 million people menstruate every day worldwide reveals the magnitude of the issue, making it clear that a system of public policies is needed to ensure access to hygiene products, adequate education on menstrual health, and sanitation and water infrastructure^(16)^. In addition, the stigma and taboos associated with menstruation, as well as the lack of adequate management of period symptoms, are issues that can only be addressed through policies that promote gender equality, inclusion, and respect for human rights^(17)^.

Thus, given the scarcity of approaches to this topic and the impacts it generates, the present study aimed to understand the perceptions of adolescent students about the menstrual cycle.

## METHOD

### Type of Study

This was a mixed-methods, descriptive, analytical, and exploratory study with a quantitative-qualitative approach defined according to the recommendations of the *Mixed Methods Appraisal Tool* (MMAT)^(18)^, since the complexity of the phenomenon studied required complementary methods^(19)^. Thus, quantitative data collection was performed first, followed by qualitative data collection.

### Location

This study was conducted at a State Technical School (ETEC) in a small municipality in the state of São Paulo, Brazil. ETEC offers five technical courses integrated with the three years of high school. In 2023, the total enrollment was 215 students.

### Selection Criteria and Definition of the Sample

The inclusion criteria were: adolescents (aged 12 to 18 years, according to the Statute of Children and Adolescents) who had already experienced menarche and were regularly enrolled in school. The exclusion criteria were: adolescents who were unable to understand the instructions or content of the research, whether due to linguistic, cognitive, or communication barriers; and adolescents who were pregnant at the time of data collection. To select the adolescents, there was initially a meeting with the ETEC’s pedagogical coordination, which listed some schedules and classes that they deemed most relevant to the research, according to the previous demands that had been made to the teachers. The adolescents from the previously listed classes were then invited to participate in a meeting held at the school, in which one of the researchers presented the project. Thus, the participants in this research were: adolescents who menstruated, whose guardians signed the Assent Form and who signed the Free and Informed Consent Form. This was therefore a convenience sample of 34 participants, i.e., all the adolescents in the classes identified by the coordination as priorities.

### Data Collection

The approach to ETEC was made by the supervising teacher who coordinated the project, directly with the school’s pedagogical coordinator, who was also responsible for the “Dignidade Íntima” (Intimate Dignity) program at ETEC. The “Dignidade Íntima” program was an initiative of the São Paulo State Government, which set aside funds for the purchase and distribution of sanitary pads in state schools.

Data collection was conducted by two researchers who had been previously trained in a research group, where they received training on the methodology of conversation circles and specific aspects of research with adolescents. Data collection was carried out in two stages: the first characterized by a quantitative approach, through the self-completion of a characterization questionnaire, constructed from the combination of the 2015 National School Health Survey (PeNSE)^(20)^ and the 2018 diversity and inclusion questionnaire^(21)^.

In the second stage of the qualitative approach, trained researchers, accompanied by the project coordinator as an observer, conducted focus groups^(22)^ with a semi-structured script (chart 1) with the adolescents who participated in the first stage and agreed to continue. The meetings took place in person at the ETEC library, during the break between the morning and afternoon shifts. Three focus groups were held between August and September 2023, with an average duration of one hour. The first took place on August 18, 2023, with first-year high school students enrolled in the technical course in Administration. The next two took place on September 1, 2023: one with second-year high school students enrolled in the technical course in Marketing and the other with third-year high school students enrolled in the technical course in Administration.

**Chart 1:** Semi-structured script – São Carlos, SP, Brazil, 2023.

1. How old were you when you had your first period, and what was it like?
2. What do you know about menstruation?
3. Do you use any type of sanitary pad or menstrual cup? How did you make that choice?
4. Who buys your sanitary pads? Do you have access to them every month?
5. Have you ever felt insecure because you were menstruating? Please tell us a little more about that.
6. Have you ever missed school because you were menstruating? Why?
7. Do you know anyone who has difficulty buying sanitary pads?
8. Have you learned about menstruation at your school? How was it addressed?
9. How does your school contribute to promoting menstrual dignity?
10. How do you think menstrual dignity should be promoted at school?

Source: Authors.

### Data Analysis

The self-completed questionnaires were collected and entered into Excel^®^ spreadsheets. The statistical program used was Stata, version 12.0, and descriptive analysis was performed to characterize the sample. All material from the discussion groups was recorded and transcribed. A textual corpus was composed by combining the responses presented, followed by processing and an initial analysis using the Interface de R pour les Analyses Multidimensionnelles de Textes et de Questionnaires (IRAMUTEQ^®^) software. Thus, the responses to the open-ended questions were organized to compose the textual corpus, which was prepared and revised in order to eliminate typing errors and standardize acronyms and expressions (preserving the same meanings). The method organizes lexical forms into classes, assigning relative importance to each of them. The occurrences of each class were considered based on statistically significant values (p < 0.05).

The findings were read and discussed using the principles of Reproductive Justice. Reproductive Justice brought to the paradigm of sexual and reproductive rights issues raised by decolonialism and intersectionality, that is, a critical perspective to counter sexist and racist patterns, among other social markers of inequality^(23)^.

### Ethical Aspects

The study was cleared by the Research Ethics Committee of the Federal University of São Carlos, under opinion number 6,308.853. All participants signed the Free and Informed Consent Form and their guardians signed the Consent Form.

## RESULTS

A total of 34 students participated in the study. Table 1 shows the profile of the responses obtained. Most identified as brown (47.06%), cisgender female (85%), and heterosexual (61.76%). Of the 34 students, 11.76% (4) reported difficulties in purchasing sanitary pads, 88.24% (30) felt insecure when menstruating, while 52.94% (18) had already missed school because they were menstruating. All interviewees reported having learned about menstruation at school.

**Table 1:** Characterization of the adolescents interviewed – São Carlos, SP, Brazil, 2023.

Variable	Proportion (n)
**Race**	
Brown	47.06% (16)
Black	11.76% (4)
White	41.18% (14)
**Age**	
15 years	17.65% (6)
16/17 years	70.59% (24)
18 years	8.82% (3)
I'd rather not answer	2.94% (1)
**Gender**	
Cis woman	85.0% (29)
I'd rather not answer	15.0% (5)
**Sexual orientation**	
Heterosexual	61.76% (21)
Bisexual	20.59% (7)
Lesbian	2.94% (1)
Demisexual	2.94% (1)
I'd rather not answer	11.77 (4)
**Do you have trouble buying sanitary pads?**	
Yes	11.76% (4)
No	88.24% (30)
**Feel insecure when you are menstruating?**	
Yes	88.24% (30)
No	8.82% (3)
No answer	2.94% (1)
**Have you ever skipped school during your period?**	
Yes	52.94% (18)
No	47.06% (16)
**Did you learned about menstruation at school?**	
Yes	100.0% (34)

Source: Authors.

With regard to the qualitative analysis of the research data, the general corpus consisted of three texts, corresponding to the transcripts of the conversation circles, separated into 106 text segments (ts), with 66ts(62.26%) and 3.552 occurrences (words, forms, or vocabulary) being used. The analyzed content was categorized into five classes: Class 1 – Challenges of Menstruation at School – with 13ts(19.7%), Class 2 – Education and Conversations about Menstruation in the Family Environment – with 12ts(18.18%), Class 3 – Support and Counseling – with 12ts(18.18%), Class 4 – Physical and Practical Difficulties of Menstruation – with 18ts(27.27%), and Class 5 – Shame and Stigmatization – with 11ts(16.67%).

**Figure 1:**
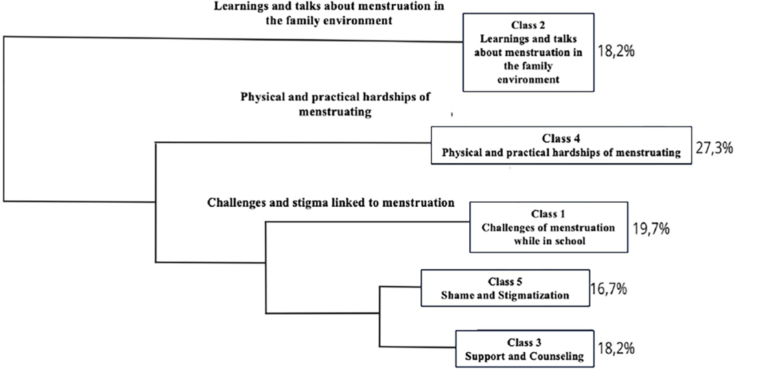
Dendrogram analysis of the textual corpus of conversation circles.

Source: Authors.

It is worth noting that these 5 classes are divided into branches, with the classes with the closest branches having greater similarity (class 3 and class 5), while the upper branches (class 2 and class 4) are more distinct from the others (Figure 1).

In this sense, we have the first theme called “Challenges and Stigma linked to Menstruation” involving classes 1 (Challenges of Menstruation while in School), 5 (Shame and Stigmatization), and 3 (Support and Counseling). The second theme is “Physical and Practical Hardships of Menstruation,” represented by class 4 (Physical and Practical Hardships of Menstruation), and finally, the third theme is “Learnings and talks about Menstruation in the Family Environment,” represented by class 2 (Learnings and talks about Menstruation in the Family Environment). Each of these classes is described and exemplified below.

### Class 1 – Challenges of Menstruation While in School

Comprises 19.7% (13 ts) of the total corpus analyzed. Consisting of words and roots in the range between x^2^ = 2.47 (period) and x^2^ = 22.23 (boy), this class is composed of words such as “school” (x^2^ = 14.54); “leave” (x^2^ = 8.23); “conversation” (x^2^ = 4.38), and “project” (x^2^ = 4.38).

From the analysis, it is possible to verify that menstruating in a school environment can be embarrassing and stigmatizing, especially when this subject is not addressed with boys, who often reproduce prejudices about the topic, as can be seen in the following excerpts:

*Boys have no idea. They make fun of it, laugh, and think that girls are stained because they want to be. It’s blood, but they react with disgust, saying it’s dirty. And we have no way of controlling that (2 MKT).*

*Sometimes this is complicated because women feel as if men are going to be ashamed of them. And this is still a perception that persists among some boys, in a way (3 ADM).*

*There was a situation where a girl menstruated without knowing it, and the blood ended up leaking. The boys started to mock her, and she left feeling very embarrassed. They kept reminding her and teasing her for about a month. It was very unpleasant, but they didn’t know much about the subject (3 ADM).*

The reports point to school as a place where the experience of menstruation can be marked by shame, fear of exposure, and psychological distress. The analysis shows that silence around the topic, especially in relation to boys, reinforces misinformation and fosters discriminatory attitudes. The absence of a universal educational approach to menstrual health contributes to an exclusionary school environment, where lack of empathy and bullying compromise the physical and emotional well-being of menstruating students. In this sense, schools, as educational spaces, need to take an active role in deconstructing stigmas and promoting gender equality.

### Class 2 – Education and Conversations About Menstruation in the Family Environment

Comprises 18.18% (12 ts) of the total corpus analyzed. Consisting of words and roots in the range between x^2^ = 2.14 (I) and x^2^ = 40.83 (mother), this class is composed of words such as “explain” (x^2^ = 24.34); “talk” (x^2^ = 5.82); “menstruation” (x^2^ = 5.3); “brother” (x^2^ = 4.97).

Through analysis, we can see the importance of menstrual education within the family environment, especially to demystify taboos, promote health and well-being, and create a safe space to discuss issues related to the menstrual cycle. In addition, talking to girls who have not yet menstruated is important to prepare them for puberty, helping to demystify the process and reduce anxiety, as well as to manage menstruation safely, as can be seen in the text fragments:

*I was embarrassed when my grandfather came home from work and my grandmother sat him down at the table, saying to everyone, “Do you know who has become a young lady?” I was 11 years old and felt very uncomfortable. Everyone stared at me, and I sat there feeling very ashamed (2 MKT).*

*My mother thinks menstruation is a big taboo at home. For example, when I got my period, she didn’t explain anything to me; she just told me to use a pad and take some medicine (3 ADM).*

*I got my period at age 12 and knew nothing about it. My cousin, who was 16, taught me how to use a pad (3 ADM).*

*My mother always talked to us about these issues since we were little. She explained that when she was young, her grandmother didn’t talk about it and, even today, she doesn’t like us to talk about it. That’s why my mother changed that reality and talked to us from an early age. So, when I got my period, I already knew a lot about it (3 ADM).*

*My mother didn’t usually explain much, but I went to live with my grandmother for a while and was away from her. It was during this period that I got my period and was desperate because I didn’t know what was happening (1 ADM).*

*I believe that, often, there is a lack of understanding on the part of the family (3 ADM).*

The statements show that the family plays a central role in shaping perceptions about menstruation and in the emotional and practical preparation for experiencing it. When the topic is treated naturally and openly, there is a reduction in anxiety and fear and greater confidence in managing the cycle. However, many reports reveal that the family environment is still marked by silence, taboos, and embarrassment, reinforcing feelings of shame and insecurity in girls. This lack of dialogue, often rooted in cultural patterns that treat menstruation as something forbidden or dirty, compromises access to essential information for care and well-being. In view of this, there is an urgent need to invest in public policies that include health education actions aimed at families, addressing menstruation in a comprehensive, affective, and intergenerational manner, in order to break cycles of misinformation and promote a culture of care, dignity, and respect from the first signs of puberty.

### Class 3 – Support and Counseling

Comprises 18.18% (12 ts) of the total corpus analyzed. Consisting of words and roots in the range between x^2^ = 2.42 (menstruation) and x^2^ = 14.14 (help), this class is composed of words such as “lecture” (x^2^ = 9.24) and “absence” (x^2^ = 4.97).

Support and counseling are essential for dealing with the menstrual cycle, especially in contexts where access to menstrual products is limited. Many girls and women face not only physical challenges, such as cramps and mood swings, but also social pressure and a lack of adequate information about their cycle. In this sense, schools play a fundamental role in reducing these challenges by providing menstrual management products, such as sanitary pads. They also provide guidance on their use and care to ensure that women do not feel isolated or embarrassed during this period, thereby reducing school absenteeism and prejudice, as can be seen below:

*This is very welcoming because we know that, if we need to, there will be someone to talk to and someone to give us sanitary pads here at school (2 MKT).*

*There was a lecture on menstruation in which boys were also included (2 MKT).*

*I believe that a lecture that includes everyone, not only women but also boys, so that they can better understand how to support girls during this period, would be important (1 ADM).*

*It is a phase in which we are very sensitive and need support during this period (3 ADM).*

*There is a project in which anyone who wanted to or faced any difficulties signed a form to receive monthly sanitary pads and wet wipes sent by the school office (1 ADM).*

*The sanitary pads come in a small box, so you can’t see what’s inside. She showed us that it was sanitary pads, because you can’t tell what it is. It is also possible to choose the quantity needed, such as 8, 16, or 46 units, and the supply occurs monthly. The material is delivered discreetly, in a corner, so that no one knows (1 ADM).*

The analysis of the reports shows the relevance of institutional support in facing the challenges associated with the menstrual cycle, especially in contexts of social and economic vulnerability. Schools emerge as a strategic and transformative space for welcoming and promoting menstrual health, whether through the provision of intimate hygiene products, such as sanitary pads and wet wipes, or through educational activities that promote information, reduce stigma, and promote gender equality. These findings reinforce the need for public policies that articulate health, education, and social assistance as a triad capable of contributing to the reduction of menstrual poverty and school retention.

### Class 4 – Physical and Practical Difficulties of Menstruation

Comprises 27.27% (18 ts) of the total corpus analyzed. Consisting of words and radicals in the range between x^2^ = 2.2 (power) and x^2^ = 14.43 (pain), this class is composed of words such as “clothing” (x^2^ = 11.35); ‘access’ (x^2^ = 11.35); “sanitary pad” (x^2^ = 5.5); “bathroom” (x^2^ = 3.82); “uncomfortable” (x^2^ = 2.46).

Through the analysis, it was possible to identify the physical and practical difficulties of the menstrual cycle, including symptoms such as cramps, fatigue, and mood swings, which can impact daily routines. In addition, the practical management of menstruation, such as the need for access to adequate products and a private place to change, can be challenging, especially in contexts where these items are scarce or where there is a lack of infrastructure. These difficulties can cause physical and emotional discomfort, leading to a negative experience of the menstrual cycle, as well as contributing significantly to school absenteeism. The following examples are provided:

*There are no decent conditions during those days; often, there are no sanitary pads or medicine to use. Many women cannot afford to buy sanitary pads, medication, or even wet wipes. We know that, in some cases, some have to use paper. We had a conversation about this, and some girls even used bread crumbs (2 MKT).*

*I believe this is poverty, because they do not have decent conditions during those days, which are already difficult to face, and they still have to go through this without any resources (2 MKT).*

*Especially women with heavy menstrual flow, who face difficulties in bathing in rural contexts, where there is often no access to water, as she mentioned (2 MKT).*

*I associated pain, care, and reprimands, because sometimes I wanted to do certain things, but I couldn’t (2 MKT).*

*I traveled to the beach a month ago, and I was in a lot of pain because of the intense cramps I have. I took medicine, but I felt bad because I couldn’t go in the water (2 MKT).*

The analysis of the statements in this class highlights the perceptions of adolescents regarding the multiple dimensions of menstrual suffering, which go beyond physical symptoms such as pain, fatigue, and mood swings, and also include material and structural barriers. The lack of sanitary pads, medication, and access to adequate bathrooms makes the menstrual cycle an experience marked by discomfort, insecurity, and exclusion, which is intensified among girls in vulnerable situations.

### Class 5 – Shame and Stigmatization

Comprises 16.67% (11 ts) of the total corpus analyzed. Consisting of words and radicals in the range between x^2^ = 2.12 (feel) and x^2^ = 45.52 (shame), this class is composed of words such as “right” (x^2^ = 15.71) and “woman” (x^2^ = 13.64).

The data show that the stigma surrounding menstruation has a profound impact on the self-esteem of people who menstruate, often leading to shame and silence around a natural process. The devaluation of menstruation can undermine confidence and hinder full participation in social and professional activities. In addition, stigma contributes to the denial of basic rights, such as access to menstrual products and education about menstrual health, perpetuating gender inequalities, as can be seen in the following excerpts:

*I believe that all women have the right to have sanitary pads at home and not face difficulties in obtaining them (2 MKT).*

*Menstruating is a negative experience because it generates fear of other people’s judgment (2 MKT).*

*In addition to embarrassment, what they were using was often not enough to contain the flow, which resulted in stains on their clothes and comments from people, increasing their discomfort (3 ADM).*

*Any small sign of blood is already a reason for comments, which generates insecurity in any woman (3 ADM).*

*Women often cannot talk about what they are feeling and experiencing because of shame and fear of being judged (1 ADM).*

The testimonies show that the shame associated with menstruation is not just an individual experience, but a reflection of how society treats the subject as taboo, viewing it as something “dirty” and “shameful,” and that people who menstruate should not talk about it. This silencing impacts emotional well-being and limits the participation of people who menstruate in different spheres of life. Overcoming this scenario requires not only the distribution of supplies, but also cultural transformation through education and the valorization of the menstrual experience as part of human health and dignity.

## DISCUSSION

This study presented the perceptions of school-aged adolescents about the menstrual cycle and how it impacts school absenteeism and the confidence of adolescents, because although most do not have difficulty acquiring menstrual management items, such as disposable sanitary pads, a large proportion do not go to school when menstruating and feel insecure during this phase of the cycle.

With regard to school absenteeism, in 2021, UNICEF and UNFPA^(24)^ conducted a survey on menstrual health and dignity through the U-Report platform throughout Brazil, to which 1.730 people, mostly between the ages of 13 and 24, voluntarily responded. Among them, 62% said they had stopped going to school or other places because of menstruation. In addition, 73% said they had felt embarrassed at school or in another public place because of menstruation. This corroborates the data from the present study, since 52.94% of students have stopped going to school while menstruating and 88.24% feel insecure during this period.

In the same study^(24)^, 71% of people who menstruate said they had never had classes, lectures, or discussion groups about menstrual care at school. In this study, however, 100% of the participants learned about menstruation at school. This significant difference may be related to the fact that the school studied participated in the Intimate Dignity Program^(25)^, which, in addition to distributing sanitary pads, provided a more welcoming space for discussion, especially for girls and adolescents enrolled in the program. It is possible that the implementation of this program provided a more favorable environment for discussion and education about menstrual health, resulting in increased awareness and learning about the topic among the participants in this study. Thus, the presence of programs for the distribution of menstrual management items, such as sanitary pads, in conjunction with other actions, seems to be a fundamental factor in addressing menstruation in schools, helping to build a culture of dialogue and information about menstrual care^(26)^.

Although 11.76% of the students in the study reported having difficulty purchasing sanitary pads, this is not the reality in Brazil when compared to other studies. According to UNICEF^(8)^, about 4 million girls do not have access to basic menstrual care items, including sanitary pads. Of these, almost 200.000 students are deprived of the minimum conditions to take care of their menstruation at school^(8)^.

Internationally, this disparity is even more pronounced. In Bangladesh, for example, more than 80% of menstruating women and girls use inappropriate materials, such as old rags, instead of appropriate sanitary products^(27)^, while in a study conducted in Ethiopia, only 35.38% of female students used sanitary pads, and among those who did not use disposable pads, 91.84% said they reused rags^(28)^. Although most discussions about period poverty focus on girls in low- and middle-income countries, period poverty also affects low-income girls in developed countries, as revealed by a study conducted in Missouri, United States, in which 64% of participants stated that they were unable to purchase menstrual products in the previous year^(29)^.

The results of this study differ from the general scenario, possibly because the school studied participated in the Intimate Dignity Program^(25)^ from 2021 to 2024, the Intimate Dignity Program^(25)^, implemented by the São Paulo State Department of Education (Seduc-SP), which provided for the availability of menstrual management items in schools, as well as promoting the training of school professionals and students on menstrual poverty.

In the same vein, with the growing importance of the issue and its social and equitable relevance, the Ministry of Education (MEC) recently launched a guidance campaign in public schools across the country on the “Menstrual Dignity Program: a cycle of respect”^(30)^. Among the program’s initiatives are the distribution of booklets, brochures, and informational videos on how to obtain free sanitary pads, which are available through the Popular Pharmacy Program and can be picked up by people between the ages of 10 and 49 (considered fertile age) who are registered in the federal government’s Single Registry for Social Programs (CadÚnico).

Although public policies for the distribution of sanitary pads are fundamental to combating menstrual poverty, menstrual health goes far beyond simple access to these items. It is an issue that involves dignity, well-being, and gender equality. In this sense, menstrual health cannot be limited to the physical aspects of this period. According to the definition developed by the Terminology Action Group of the Global Menstrual Collective, menstrual health represents a state of complete physical, mental, and social well-being, going beyond the absence of diseases or illnesses related to the menstrual cycle^(31)^.

Within this comprehensive concept, the Global Menstrual Collective itself emphasizes the importance of ensuring access to reliable information about the menstrual cycle, appropriate for each age group, in addition to promoting self-care and hygiene practices that respect personal preferences, ensuring comfort, privacy, and safety. The collective also emphasizes the need to offer timely diagnosis and treatment for discomfort and disorders related to the menstrual cycle, including pain relief. In addition, it highlights the importance of ensuring a welcoming environment, free from judgment, discrimination, or violence related to menstruation, allowing menstruating individuals to decide, without restrictions or coercion, to participate fully in civil, cultural, economic, social, and political spheres at all stages of their menstrual cycle^(32)^.

Understanding the menstrual cycle as a gender issue is essential, as males who do not experience the menstrual cycle are affected by the social, cultural, and economic aspects of menstruation^(33)^. A study that sought to understand the influence of gender on the social representation of menstruation found that among young people, men tend to refer to menstruation as “dirty” and that it makes women more fragile and susceptible to emotions^(34)^.

In the school environment, this issue is even more acute. Boys’ lack of knowledge about the menstrual cycle often results in bullying and derogatory comments, which intensifies girls’ feelings of shame. According to a survey conducted in a school environment, 13% of girls have experienced menstrual teasing and more than 80% fear being teased, especially by male classmates. Girls cope by reducing their school attendance, participation, and concentration in the classroom during their periods^(35)^.

Given this scenario, it is essential to include the male perspective on issues related to menstruation to promote effective change. Education about the menstrual cycle should begin in school, addressing both boys and girls. People who do not menstruate express interest in learning about the topic^(36)^, and including this issue as part of sex education and life skills should not be just a formality, but rather a concrete action. By educating boys about menstrual management, they will grow up more informed and able to contribute to reducing menstrual stigma, creating more inclusive and healthy school environments for everyone.

The main sources of information about menarche for adolescents are mostly their mothers (39.7%), followed by friends (26.6%) and teachers (21.8%)^(26)^. The attitudes and beliefs that families have about menstruation significantly influence how girls and boys understand and deal with the menstrual cycle.

One study found that the likelihood of good menstrual hygiene management practices was higher among those who had knowledge about menstrual hygiene before menarche and had discussed the menstrual cycle with their parents (36). However, many girls reported that their families, especially their mothers or fathers, were not open to discussing these issues, making menstruation a taboo subject at home.

This lack of open dialogue on topics such as menstrual flow variation, menstrual management items, and associated symptoms negatively impacted these girls’ menstrual experience, especially in early adolescence, when they are still adjusting to their new bodily reality^(36)^.

In this context, although menstrual dignity has gained more attention and generated public policies, such as those mentioned above, studies reveal that there are still gaps to be filled. It is essential to consider the experience of other menstruating bodies, in addition to cisgender girls and women^(37)^, as well as an intersectional perspective on the issue, since menstrual poverty affects more intensely residents of the periphery and black women^(38)^. In addition, there is an urgent need for more research in Brazil that addresses school-age adolescents, as well as explores different regions and cultural realities of the country, allowing for a more comprehensive and equitable perspective on the topic. Future studies will also be essential to analyze the impact of the Menstrual Dignity Program, which was recently implemented, enabling its improvement.

As seen in this research, there remains a significant gap in the welcoming offered by these policies for the distribution of menstrual management items, especially when it comes to recognizing and addressing menstrual pain that affects so many people who menstruate. This daily reality is often underestimated and trivialized, with the associated difficulties being naturalized rather than adequately considered and addressed^(39,40)^.

Limitations of this study include the failure to obtain a larger number of responses to the questionnaire and subsequent participation in the discussion group, which affected the power of inference in the quantitative analysis. In addition, one of the gaps in the research was not involving male adolescents in the focus groups, which could have contributed to a broader and more inclusive understanding of menstruation, challenging stigmas and promoting greater awareness among all genders. These limitations reinforce the need for future studies that broaden the scope of participants and address the experiences of different gender identities for a more complete and inclusive view.

## CONCLUSION

This research highlighted how the menstrual cycle directly and indirectly affects the daily, school, and emotional lives of adolescents, generating insecurity, school absenteeism, and possible difficulty in accessing menstrual management products. Among the main results, it is noted that more than half of the adolescents have already stopped going to school when menstruating and reported feeling insecure during this period. Although school is a central source of learning about the topic, issues such as shame and stigmatization still persist, especially among male adolescents. By recounting the experiences of adolescents, the study contributes to the visibility of the menstrual cycle as an important issue, pointing to the need for educational and family interventions that broaden dialogue and support, promoting greater welcoming and understanding of menstrual challenges. These findings reinforce the need for intersectoral actions that integrate education, health, and social assistance to ensure menstrual dignity, combat stigma, and promote more inclusive and equitable school environments.

## DATA AVAILABILITY

The entire dataset supporting the results of this study is available upon request to the corresponding author.
