# Supplementary material for: Menstrual health: an analysis of experiences among school-aged adolescents
Source: Rev Esc Enferm USP. 2026 Jan 19;59:e20250003. doi: 10.1590/1980-220X-REEUSP-2025-0003en (PMC12834388; doi:10.1590/1980-220X-REEUSP-2025-0003en)
Supplement: Translation

# Translation: Saúde menstrual: uma análise de experiências entre adolescentes escolares

**DOI:** 10.1590/1980-220X-REEUSP-2025-0003pt

**Authors:** Jennifer Caroline de Oliveira Adomaitis, Natália Sevilha Stofel, Maria Gabriele Formis Silva, Vivian Campos Santos, Flávio Adriano Borges, Diene Monique Carlos, Bruna Luana de Souza Farias, Márcia Niituma Ogata, Juliana Cristina dos Santos Monteiro

**Affiliations:** 1 Universidade Federal de São Carlos, Departamento de Enfermagem, São Carlos, SP, Brasil.; 2 Universidade de São Paulo, Escola de Enfermagem de Ribeirão Preto, Ribeirão Preto, SP, Brasil.; 3 Universidade Federal de São Carlos, Programa de Pós-Graduação em Enfermagem, São Carlos, SP, Brasil.

**Keywords:** Adolescente, Menstruação;, Instituições Acadêmicas

## Abstract

**Objetivo:**

Compreender as percepções de adolescentes escolares sobre o ciclo menstrual.

**Método:**

Tratou-se de uma pesquisa de métodos mistos, exploratória, em uma Escola Técnica Estadual, no interior de São Paulo. Foi realizada análise estatística descrita para os dados quantitativos e o uso do software Iramuteq, para os dados qualitativos. Os achados foram confrontados com os princípios da Justiça Reprodutiva.

**Resultados:**

Participaram desta pesquisa 34 adolescentes. Constatou-se que 17% tinham dificuldades para comprar absorventes, 88% sentia-se insegura quando estava menstruada, 53% já deixou de ir à escola por estar menstruada. Todas afirmaram ter aprendido sobre menstruação na escola. Emergiram cinco classes temáticas: 1) Desafios da Menstruação na Escola; 2) Educação e Conversas sobre Menstruação no Ambiente Familiar; 3) Apoio e Aconselhamento; 4) Dificuldades Físicas e Práticas da Menstruação; 5) Vergonha e Estigmatização.

**Conclusão:**

A pesquisa revela que a menstruação impacta significativamente a vida das adolescentes, gerando insegurança e ausência nas aulas. A escola desempenha um papel significativo na disseminação de informações sobre o tema, os dados obtidos apontam a necessidade de ampliar o debate e reduzir estigmas.

## INTRODUÇÃO

A menstruação, embora seja um processo fisiológico, é frequentemente acompanhada não apenas por desconfortos físicos e mudanças emocionais, mas também por estigmas, sendo considerada um tabu. Em diversas culturas, há uma tendência a associar a menstruação a algo impuro ou negativo^(1)^. O patriarcado, enquanto normalizador dos corpos e comportamentos sociais, ao incidir sobre o ciclo menstrual, produz e reproduz desinformações, criando olhares pejorativos, colocando a menstruação em um lugar que desperta nojo^(2)^. Ter essa visão associada ao corpo biologicamente feminino, segrega ainda mais as pessoas que menstruam^(3)^.

A pobreza menstrual refere-se à dificuldade ou falta de acesso a produtos de manejo essenciais durante o período menstrual, bem como à indisponibilidade de locais adequados para utilizálos. Isso inclui a ausência de serviços básicos de saneamento e a limitação no acesso a informações sobre a menstruação^(4)^. Em essência, pobreza menstrual é um grave problema social que reflete as desigualdades sociais e de gênero, especialmente ao compararmos o alto custo dos produtos de manejo menstrual com outros itens de cuidados pessoais^(5)^. A menstruação é invisibilizada, fazendo com que não seja discutida, e os problemas não são observados, no entanto eles não desaparecem e atingem de forma intensa as pessoas que são mais vulnerabilizadas^(6)^.

Além da dificuldade de acesso a produtos e da falta de infraestrutura, é importante considerar que a menstruação envolve uma série de necessidades relacionadas à saúde física, emocional e social. O conceito de saúde menstrual é definido como um estado de bem-estar físico, mental e social em relação ao ciclo menstrual, e não apenas a ausência de doenças ou enfermidades. Essa abordagem amplia o olhar para além das condições materiais, incluindo também o direito à informação sobre a menarca, ao acesso a espaços seguros e privados para cuidar da higiene menstrual e à possibilidade de participação plena em atividades educacionais, sociais e profissionais durante o período menstrual^(7)^.

Reconhecer a saúde menstrual como um direito fundamental implica considerar que tanto a pobreza menstrual quanto a falta de cuidados adequados à saúde menstrual contribuem para impactos negativos na vida das pessoas que menstruam, entre os impactos gerados, um dos mais significativos é o absenteísmo escolar. Segundo dados do estudo do Fundo de Populações das Nações Unidas (UNFPA), uma em cada 10 meninas faltam à escola quando está menstruada^(8)^. No Brasil, segundo dados do guia de implementação do Programa de Dignidade Menstrual, uma em cada quatro meninas se ausenta da escola quando está menstruada por não conseguir comprar absorventes; cerca de 4 milhões de meninas sofrem com pelo menos uma privação de recursos nas escolas (acesso a absorventes e/ou instalações básicas, tais como banheiros e sabonetes), além disso, somente 20% das alunas sentiam-se bem informadas quando vivenciaram sua primeira menstruação^(9)^.

A falta de informação e acesso a produtos de manejo menstrual, aliada a preconceitos e dificuldades, gera desconforto, constrangimento e até bullying, resultando na exclusão das meninas de diversas atividades cotidianas e impactando negativamente seu rendimento escolar e profissional, em comparação com pessoas que não menstruam^(10)^. Além disso, os estigmas fazem com que as meninas evitem trocar o absorvente na escola, o que compromete sua saúde física. A “etiqueta menstrual” também reforça a ideia de que não se deve falar abertamente sobre menstruação, o que prejudica a autoestima e a confiança das meninas. Esse estigma é fundamentado na concepção de que o sangue menstrual é sujo ou impuro, algo a ser evitado^(11)^.

Outros fatores que contribuem para o absenteísmo são os sintomas físicos associados à menstruação, como a dismenorreia, que se caracteriza por cólicas menstruais intensas de origem uterina, um dos estudos indica que essa condição afeta até 95% das mulheres que menstruam^(12)^; a Síndrome Pré-Menstrual, caracterizada por um conjunto de manifestações físicas, emocionais e comportamentais que surgem durante a fase lútea do ciclo menstrual (geralmente na semana anterior à menstruação) e tendem a desaparecer com o início do sangramento menstrual^(13)^.

Além dos sintomas físicos, a menstruação também gera impactos emocionais significativos. Um estudo transversal conduzido no Japão em 2022, com 198 estudantes do ensino médio, revelou que os sintomas menstruais, como dor intensa (dismenorréia), impacto nas atividades cotidianas, menstruação inesperada e necessidade frequente de medicação analgésica, estavam fortemente associados a escores mais baixos de qualidade de vida, especialmente nos aspectos relacionados à saúde mental^(14)^. Os impactos desses sintomas são profundos e podem comprometer a qualidade de vida, interferindo nas atividades diárias e levando à exclusão escolar, profissional e social. Apesar de sua alta prevalência, muitas pessoas enfrentam um suporte de saúde insuficiente ou desumano^(15)^.

A realidade de que cerca de 800 milhões de pessoas menstruam todos os dias em todo o mundo revela a magnitude da questão, tornando evidente que é necessário um sistema de políticas públicas que garanta o acesso a produtos de higiene, educação adequada sobre a saúde menstrual, e infraestrutura de saneamento e água^(16)^. Além disso, o estigma e os tabus associados à menstruação, bem como a falta de manejo adequado para a sintomatologia do período, são questões que só podem ser abordadas por meio de políticas que promovam a igualdade de gênero, a inclusão e o respeito pelos direitos humanos^(17)^.

Assim, diante da escassez da abordagem da temática e os impactos que ela gera, o presente trabalho teve como objetivo compreender as percepções de adolescentes escolares sobre o ciclo menstrual.

## MÉTODO

### Tipo de Estudo

Tratou-se de uma pesquisa de métodos mistos, descritivo, analítico e exploratório de abordagem quanti-qualitativa definida seguindo a recomendação da *Mixed Methods Appraisal Tool* (MMAT)^(18)^, uma vez que a complexidade do fenômeno estudado requereu complementaridade dos métodos^(19)^. Assim, foi primeiramente realizada a coleta de dados quantitativa e na sequência, uma qualitativa.

### Local

O presente estudo foi realizado em uma Escola Técnica Estadual (ETEC) em um município de pequeno porte, no interior do estado de São Paulo, Brasil. A ETEC oferece cinco cursos técnicos integrados aos três anos do ensino médio, em 2023, o total de matrículas era de 215 estudantes.

### Critérios de Seleção e Definição da Amostra

Foram critérios de inclusão: adolescentes (com idade de 12 a 18 anos completos, segundo o Estatuto da Criança e do Adolescente) que já tivessem passado pela menarca e regularmente matriculadas na escola. Como critérios de exclusão foram elencados: adolescentes que não conseguissem compreender as instruções ou o conteúdo da pesquisa, seja por barreiras linguísticas, cognitivas ou de comunicação; e adolescentes que, no momento da coleta, estivessem gestantes. Para a seleção das adolescentes, houve inicialmente uma reunião com a coordenação pedagógica da ETEC, que elencou alguns horários e turmas que julgaram que a pesquisa era mais pertinente, de acordo com as demandas prévias que chegavam aos docentes. As adolescentes das turmas previamente elencadas foram então convidadas a participar em um encontro realizado na escola, no qual uma das pesquisadoras apresentou o projeto. Assim, foram participantes desta pesquisa: adolescentes que menstruavam, cujos responsáveis assinaram o Termo de Assentimento e elas assinaram o Termo de Consentimento Livre e Esclarecido. Tratando-se assim, de amostra por conveniência que contou com 34 participantes, ou seja, todas as adolescentes das turmas apontadas pela coordenação como prioritárias.

### Coleta de Dados

A aproximação com a ETEC foi feita pela professora orientadora que coordenava o projeto, diretamente com a coordenadora pedagógica da escola, que também era a responsável pelo programa “Dignidade Íntima” na ETEC. O programa “Dignidade Íntima” era uma iniciativa do governo do Estado de São Paulo, que reservava uma verba para compras e distribuição de absorventes menstruais nas escolas estaduais.

A coleta de dados foi conduzida por duas pesquisadoras previamente treinadas em um grupo de pesquisa, onde receberam formação sobre a metodologia das rodas de conversa e aspectos específicos da pesquisa com adolescentes. A coleta de dados foi realizada em duas etapas: a primeira caracterizada pela abordagem quantitativa, através do autopreenchimento de um questionário de caracterização, construído a partir da junção do questionário Pesquisa Nacional de Saúde do Escolar (PeNSE) de 2015^(20)^ e o questionário diversidade e inclusão de 2018^(21)^.

Na segunda etapa de abordagem qualitativa, as pesquisadoras treinadas, acompanhadas pela coordenadora do projeto na função de observadora, conduziram grupos focais^(22)^ com roteiro semiestruturado (Quadro 1) com as adolescentes que participaram da primeira etapa e concordaram em prosseguir. Os encontros se deram de maneira presencial na biblioteca da ETEC, no intervalo entre o turno matutino e vespertino. Foram realizados três grupos focais entre os meses de agosto e setembro de 2023, com duração média de uma hora. A primeira ocorreu em 18 de agosto de 2023, com estudantes do primeiro ano do ensino médio integrado ao curso técnico em Administração. As duas seguintes aconteceram em 1º de setembro de 2023: uma com alunas do segundo ano do ensino médio integrado ao curso de técnico em Marketing e a outra com estudantes do terceiro ano do ensino médio integrado ao curso técnico em Administração.

**Quadro 1:** Roteiro semiestruturado – São Carlos, SP, Brasil, 2023.

1. Com quantos anos você teve a sua primeira menstruação e como foi?
2. O que você sabe sobre menstruação?
3. Você utiliza algum tipo de absorvente ou coletor menstrual? Como foi essa escolha?
4. Quem compra seu absorvente? Você tem acesso todos os meses a eles?
5. Você já se sentiu insegura/o/e por estar menstruada/e/o? Conte um pouco mais sobre isso, por favor.
6. Você já deixou de ir à escola por estar menstruada/o/e? Qual o motivo?
7. Você conhece alguém que tenha dificuldade para comprar absorventes?
8. Você já aprendeu sobre menstruação em sua escola? E como foi abordado?
9. Como a sua escola contribui na promoção da dignidade menstrual?
10. Como você acredita que deva ser a promoção da dignidade menstrual na escola?

Fonte: Autores.

### Análise dos Dados

Os questionários auto-preenchidos foram recolhidos e digitados em planilhas do software Excel^®^. O programa estatístico utilizado foi o Stata, versão 12.0, sendo realizada a análise descritiva para caracterizar a amostra. Todo o material das rodas de conversa foi gravado e transcrito. Foi composto um corpus textual por meio da junção das respostas apresentadas, seguindo com seu processamento e uma primeira análise por meio do software Interface de R pour les Analyses Multidimensionnelles de Textes et de Questionnaires (IRAMUTEQ^®^). Assim, as respostas das questões abertas foram organizadas de forma a compor o corpus textual, que foi preparado e revisado com o intuito de eliminar equívocos de digitação e padronização de siglas e expressões (preservando os mesmos significados). O método organiza as formas lexicais em classes, atribuindo importância relativa a cada uma delas. As ocorrências de cada uma das classes foram consideradas a partir de valores estatisticamente significantes (p < 0,05).

Os achados obtidos foram lidos e discutidos através dos princípios da Justiça Reprodutiva. A Justiça Reprodutiva trouxe ao paradigma dos direitos sexuais e reprodutivos questões colocadas pelo decolonialismo e pela interseccionalidade, ou seja, uma perspectiva crítica para contrapor padrões sexistas, racistas, entre outros marcadores sociais de desigualdade^(23)^.

### Aspectos Éticos

O estudo foi aprovado pelo Comitê de Ética em Pesquisa da Universidade Federal de São Carlos, sob número de parecer 6.308.853. Todas as participantes assinaram o Termo de Consentimento Livre e Esclarecido e seus responsáveis o Termo de Assentimento.

## RESULTADOS

No total, 34 estudantes participaram da pesquisa. Na Tabela 1 é apresentado o perfil das respostas obtidas. A maioria se declarou parda (47,06%), feminina cisgênero (85%) e heterossexual (61,76%). Das 34 estudantes, constatou-se que 11,76% (4) das participantes possuem dificuldades para comprar absorventes, 88,24% (30) se sente insegura quando está menstruada, enquanto que 52,94% (18) já deixou de ir à escola por estar menstruada. Todas as entrevistadas afirmaram ter aprendido sobre menstruação na escola.

**Tabela 1:** Caracterização das adolescentes entrevistadas – São Carlos, SP, Brasil, 2023.

Variável	Proporção (n)
**Raça/Cor**	
Parda	47,06% (16)
Preta	11,76% (4)
Branca	41,18% (14)
**Idade**	
15 anos	17,65% (6)
16/17 anos	70,59% (24)
18 anos	8,82% (3)
Prefiro não responder	2,94% (1)
**Gênero**	
Mulher Cis	85,0% (29)
Prefiro não responder	15,0% (5)
**Orientação Sexual**	
Heterossexual	61,76% (21)
Bissexual	20,59% (7)
Lésbica	2,94% (1)
Demissexual	2,94% (1)
Prefiro não responder	11,77 (4)
**Possui dificuldades para comprar absorventes?**	
Sim	11,76% (4)
Não	88,24% (30)
**Sente insegurança quando está menstruada?**	
Sim	88,24% (30)
Não	8,82% (3)
Não respondeu	2,94% (1)
**Já deixou de ir à escola no período menstrual?**	
Sim	52,94% (18)
Não	47,06% (16)
**Aprendeu sobre menstruação na escola?**	
Sim	100,0% (34)

Fonte: Autores.

No que diz respeito à análise qualitativa dos dados da pesquisa, o corpus geral foi constituído por três textos, que correspondem às transcrições das rodas de conversa, separados em 106 segmentos de texto (st), com aproveitamento de 66 st (62,26%) e 3552 ocorrências (palavras, formas ou vocábulos). O conteúdo analisado foi categorizado em cinco classes: Classe 1 – Desafios da Menstruação na Escola – com 13 st (19,7%), Classe 2 – Educação e Conversas sobre Menstruação no Ambiente Familiar – com 12 st (18,18%), Classe 3 – Apoio e Aconselhamento – com 12 st (18,18%), Classe 4 – Dificuldades Físicas e Práticas da Menstruação – com 18 st (27,27%) e Classe 5 – Vergonha e Estigmatização – com 11 st (16,67%).

Vale ressaltar que essas 5 classes se encontram divididas por ramificações, sendo que as classes com ramificações mais próximas possuem maior similaridade (classe 3 e classe 5), enquanto que as ramificações superiores (classe 2 e classe 4) são mais distintas das demais (Figura 1).

**Figura 1:** Dendograma de análise do corpus textual das rodas de conversa.

Fonte: Autores.

Nesse sentido, temos o primeiro tema denominado “Desafios e Estigma Relacionados à Menstruação” envolvendo as classes 1 (Desafios da Menstruação na Escola), 5 (Vergonha e Estigmatização) e 3 (Apoio e Aconselhamento). O segundo tema é “Dificuldades Físicas e Práticas da Menstruação”, representado pela classe 4 (Dificuldades Físicas e Práticas da Menstruação) e por fim o terceiro tema é “Educação e Conversas sobre Menstruação no Ambiente Familiar”, representado pela classe 2 (Educação e Conversas sobre Menstruação no Ambiente Familiar). A seguir estão descritas e exemplificadas cada uma dessas classes.

### Classe 1 – Desafios da Menstruação na Escola

Compreende 19,7% (13 st) do corpus total analisado. Constituída por palavras e radicais no intervalo entre x^2^ = 2,47 (período) e x^2^ = 22,23 (menino), essa classe é composta por palavras como “escola” (x^2^ = 14,54); “sair” (x^2^ = 8,23); “conversa” (x^2^ = 4,38) e projeto (x^2^ = 4,38).

A partir da análise é possível verificar que menstruar em ambiente escolar pode ser constrangedor e estigmatizante, especialmente quando esse assunto não é abordado com os meninos, que muitas vezes reproduzem preconceitos a respeito da temática, como se pode perceber por meio dos fragmentos:

*O menino não tem noção. Ele tira sarro, dá risada e acha que a menina está manchada porque quer. É sangue, mas eles reagem com nojo, dizendo que é sujo. E a gente não tem como controlar isso (2 MKT).*

*Às vezes, isso é complicado porque a mulher se sente como se o homem fosse sentir vergonha dela. E essa ainda é uma percepção que persiste entre alguns meninos, de certo modo (3 ADM).*

*Houve uma situação em que uma menina menstruou sem saber, e o sangue acabou vazando. Os meninos passaram a zombar dela, e ela foi embora muito constrangida. Eles continuaram lembrando e provocando por cerca de um mês. Foi algo bastante desagradável, mas eles não tinham muito conhecimento sobre o assunto (3 ADM).*

Os relatos apontam a escola como um espaço onde a experiência da menstruação pode ser atravessada por vergonha, medo de exposição e sofrimento psíquico. A análise demonstra que o silêncio em torno do tema, especialmente em relação aos meninos, reforça a desinformação e fomenta atitudes discriminatórias. A ausência de uma abordagem educativa universal sobre saúde menstrual contribui para um ambiente escolar excludente, onde a falta de empatia e o bullying comprometem o bem-estar físico e emocional dos estudantes que menstruam. Nesse sentido, a escola, enquanto espaço formador,precisa assumir um papel ativo na desconstrução de estigmas e na promoção da equidade de gênero.

### Classe 2 – Educação e Conversas Sobre Menstruação no Ambiente Familiar

Compreende 18,18% (12 st) do corpus total analisado. Constituída por palavras e radicais no intervalo entre x^2^ = 2,14 (eu) e x^2^ = 40,83 (mãe), essa classe é composta por palavras como “explicar” (x^2^ = 24,34); “falar” (x^2^ = 5,82); “menstruação” (x^2^ = 5,3); “irmão” (x^2^ = 4,97).

Por meio da análise, percebe-se a importância da educação menstrual dentro do ambiente familiar, especialmente para desmistificar tabus, promover a saúde e bem-estar, e criar um espaço seguro para discutir questões relacionadas ao ciclo menstrual. Além disso, conversar com meninas que ainda não menstruaram é importante para prepará-las para a puberdade, ajudando a desmistificar o processo e reduzir a ansiedade, assim como fazer o manejo seguro da menstruação, como se pode perceber por meio dos fragmentos de texto:

*Passei vergonha quando meu avô chegou do trabalho e minha avó o sentou à mesa, dizendo para todos: “Já sabe quem ficou mocinha?” Eu tinha 11 anos e fiquei muito constrangida. Todos ficaram olhando, e eu fiquei sentada, me sentindo bastante envergonhada (2 MKT).*

*Minha mãe acha que a menstruação é um grande tabu em casa. Por exemplo, quando eu menstruei, ela não me explicou nada; apenas disse para eu usar o absorvente e tomar um remédio (3 ADM).*

*Menstruei aos 12 anos e não sabia nada sobre o assunto. Quem me ensinou a colocar absorvente foi minha prima, que tinha 16 anos (3 ADM).*

*Minha mãe sempre conversou com a gente sobre esses assuntos desde que éramos pequenas. Ela explicou que, quando era jovem, a avó dela não falava sobre isso e, até hoje, não gosta que conversemos a respeito. Por isso, minha mãe mudou essa realidade e conversava conosco desde cedo. Assim, quando menstruei, já sabia bastante sobre o assunto (3 ADM).*

*Minha mãe não costumava explicar muito, mas eu fui morar com minha avó por um tempo e fiquei afastada dela. Foi nesse período que menstruei e fiquei desesperada, pois não sabia o que estava acontecendo (1 ADM).*

*Acredito que, muitas vezes, falta compreensão por parte da família (3 ADM).*

As falas evidenciam que a família exerce um papel central na construção das percepções sobre a menstruação e no preparo emocional e prático para vivenciá-la, sendo que, quando o tema é tratado com naturalidade e abertura, há redução da ansiedade, do medo e maior segurança no manejo do ciclo. Contudo, muitos relatos revelam que o ambiente familiar ainda está marcado por silêncios, tabus e constrangimentos, reforçando sentimentos de vergonha e insegurança nas meninas. Essa ausência de diálogo, muitas vezes enraizada em padrões culturais que tratam a menstruação como algo proibido ou sujo, compromete o acesso a informações essenciais para o cuidado e o bem-estar. Diante disso, torna-se urgente o investimento em políticas públicas que incluam ações de educação em saúde voltadas às famílias, abordando a menstruação de forma integral, afetiva e intergeracional, de modo a romper ciclos de desinformação e promover uma cultura do cuidado, da dignidade e do respeito desde os primeiros sinais da puberdade.

### Classe 3 – Apoio e Aconselhamento

Compreende 18,18% (12 st) do corpus total analisado. Constituída por palavras e radicais no intervalo entre x^2^ = 2,42 (menstruação) e x^2^ = 14,14 (ajudar), essa classe é composta por palavras como “palestra” (x^2^ = 9,24) e “falta” (x^2^ = 4,97).

O apoio e o aconselhamento são fundamentais para lidar com o ciclo menstrual especialmente em contextos onde o acesso a produtos menstruais é limitado. Muitas meninas e mulheres enfrentam não apenas desafios físicos, como cólicas e mudanças de humor, mas também a pressão social e a falta de informações adequadas sobre seu ciclo. Nesse sentido, a escola possui um papel fundamental para reduzir esses desafios, por meio da disponibilização de produtos de manejo menstrual, como absorventes. Assim como, dar orientação sobre seu uso e cuidados para garantir que as mulheres não se sintam isoladas ou constrangidas durante esse período, reduzindo até mesmo o absenteísmo escolar e o preconceito, como pode se ver a seguir:

*Isso é muito acolhedor, porque sabemos que, se precisarmos, haverá alguém com quem conversar e de quem receber absorventes aqui na escola (2 MKT).*

*Houve uma palestra sobre menstruação na qual os meninos também foram incluídos (2 MKT).*

*Acredito que uma palestra que inclua todas as pessoas, não só as mulheres, mas também os meninos, para que eles entendam melhor como apoiar as meninas durante esse período, seria importante (1 ADM).*

*É uma fase em que estamos muito sensíveis e precisamos de apoio nesse período (3 ADM).*

*Há um projeto em que, quem quisesse ou enfrentasse alguma dificuldade, assinava um termo para receber mensalmente absorventes e lenços umedecidos enviados pela secretaria (1 ADM).*

*Os absorventes vêm em uma caixinha embalada, de forma que não é possível identificar o que tem dentro. Ela nos mostrou que era absorvente, pois não dá para saber o que é. Também é possível escolher a quantidade necessária, como 8, 16 ou 46 unidades, e o fornecimento ocorre mensalmente. O material é entregue discretamente, em um cantinho, para que ninguém saiba (1 ADM).*

A análise dos relatos mostra a relevância do apoio institucional no enfrentamento dos desafios associados ao ciclo menstrual, especialmente em contextos de vulnerabilidade social e econômica. A escola emerge como espaço estratégico e transformador para acolhimento e promoção da saúde menstrual, seja por meio da oferta de produtos de higiene íntima, como absorventes e lenços umedecidos, seja pela realização de atividades educativas que promovam informação, reduzam o estigma e promovam a equidade de gênero. Esses achados reforçam a necessidade de políticas públicas que articulem saúde, educação e assistência social, como uma tríade capaz de contribuir para a redução da pobreza menstrual e permanência escolar.

### Classe 4 – Dificuldades Físicas e Práticas da Menstruação

Compreende 27,27% (18 st) do corpus total analisado. Constituída por palavras e radicais no intervalo entre x^2^ = 2,2 (poder) e x^2^ = 14,43 (dor), essa classe é composta por palavras como “roupa” (x^2^ = 11,35); “acesso” (x^2^ = 11,35); “absorvente” (x^2^ = 5,5); “banheiro” (x^2^ = 3,82); “desconfortável” (x^2^ = 2,46).

Por meio da análise foi possível identificar as dificuldades físicas e práticas do ciclo menstrual, incluindo sintomas como cólicas, fadiga, e alterações de humor, que podem impactar a rotina diária. Além disso, a gestão prática da menstruação, como a necessidade de ter acesso a produtos adequados e um local privativo para a troca, pode ser um desafio, especialmente em contextos onde esses itens são escassos ou onde há falta de infraestrutura. Essas dificuldades podem gerar desconforto físico e emocional, levando a uma experiência negativa do ciclo menstrual, além de contribuir significativamente para o absenteísmo escolar, a seguir exemplos estão colocados:

*Não há condições dignas nesses dias, muitas vezes, não há absorvente ou remédio para usar. Muitas mulheres não conseguem comprar absorventes, remédios ou até lenços umedecidos. Sabemos que, em alguns casos, algumas têm que usar papel. Tivemos uma conversa sobre isso, e algumas meninas chegaram a usar miolo de pão (2 MKT).*

*Acredito que isso é pobreza, pois elas não têm condições dignas nesses dias que já são difíceis de enfrentar, e ainda precisam passar por isso sem nenhum recurso (2 MKT).*

*Principalmente mulheres com fluxo menstrual intenso, que enfrentam dificuldades para tomar banho em contextos rurais, onde muitas vezes não há acesso à água, como ela mencionou (2 MKT).*

*Eu associei dor, cuidado e repreensão, porque às vezes eu queria fazer certas coisas, mas não podia (2 MKT).*

*Viajei para a praia há um mês, e estava com muita dor por causa das cólicas intensas que tenho. Tomei remédio, mas me senti mal porque não pude entrar na água (2 MKT).*

A análise das falas dessa classe evidencia as percepções das adolescentes frente às múltiplas dimensões do sofrimento menstrual, que vão além dos sintomas físicos como dor, fadiga e alterações de humor, incluindo ainda barreiras materiais e estruturais. A ausência de absorventes, medicamentos e acesso a banheiros adequados torna o ciclo menstrual uma experiência marcada por desconforto, insegurança e exclusão, se intensificando entre meninas em situação de vulnerabilidade.

### Classe 5 – Vergonha e Estigmatização

Compreende 16,67% (11 st) do corpus total analisado. Constituída por palavras e radicais no intervalo entre x^2^ = 2,12 (sentir) e x^2^ = 45,52 (vergonha), essa classe é composta por palavras como “direito” (x^2^ = 15,71) e “mulher” (x^2^ = 13,64).

Os dados mostram que o estigma em torno da menstruação tem um impacto profundo na autoestima das pessoas que menstruam, muitas vezes levando à vergonha e ao silêncio em torno de um processo natural. A desvalorização da menstruação pode minar a confiança e dificultar a participação plena em atividades sociais e profissionais. Além disso, o estigma contribui para a negação de direitos básicos, como o acesso a produtos menstruais e à educação sobre saúde menstrual, perpetuando desigualdades de gênero, como pode se ver nos trechos a seguir:

*Acredito que todas as mulheres têm o direito de ter absorventes em casa e não enfrentar dificuldades para obtê-los (2 MKT).*

*Menstruar é uma experiência negativa porque gera medo do julgamento das outras pessoas (2 MKT).*

*Além do constrangimento, muitas vezes o que elas estavam usando não era suficiente para conter o fluxo, o que resultava em manchas na roupa e nos comentários das pessoas, aumentando o desconforto (3 ADM).*

*Qualquer pequeno sinal de sangue já é motivo de comentários, o que gera insegurança em qualquer mulher (3 ADM).*

*A mulher muitas vezes não pode falar sobre o que está sentindo e vivenciando por vergonha e medo de ser julgada (1 ADM).*

Os depoimentos evidenciam que a vergonha associada à menstruação não é apenas uma experiência individual, mas um reflexo de como a sociedade trata o tema como tabu, sendo vista como algo “sujo” e “vergonhoso”, sendo que as pessoas que menstruam não devem falar sobre o tema. Esse silenciamento impacta o bem-estar emocional e limita a participação das pessoas que menstruam em diferentes esferas da vida. Superar esse cenário exige não apenas distribuição de insumos, mas também transformação cultural por meio da educação e da valorização da experiência menstrual como parte da saúde e dignidade humana.

## DISCUSSÃO

O presente estudo trouxe as percepções de adolescentes escolares sobre o ciclo menstrual e como ele impacta no absenteísmo escolar e na confiança das adolescentes, pois embora a maioria não tenha dificuldade em adquirir itens de gestão menstrual, como absorventes descartáveis, uma grande parte não vai à escola quando menstruada e se sente insegura nesta fase do ciclo.

No que se refere ao absenteísmo escolar, em 2021, a UNICEF e a UNFPA^(24)^ realizaram uma enquete sobre saúde e dignidade menstrual, por meio da plataforma U-Report, em todo o Brasil, na qual responderam voluntariamente 1.730 pessoas, a maioria entre 13 e 24 anos de idade. Entre elas, 62% afirmam que já deixaram de ir à escola ou outros lugares por causa da menstruação. Além disso, 73% disseram que já sentiram constrangimento na escola ou em outro local público por causa da menstruação. Isso corrobora os dados da presente pesquisa, visto que 52,94% estudantes deixaram de ir à escola enquanto menstruadas e 88,24% se sentiram inseguras durante esse período.

Nessa mesma pesquisa^(24)^, 71% das pessoas que menstruam disseram que nunca tiveram aulas, palestras ou rodas de conversa sobre cuidados na menstruação na escola. Enquanto que nesta pesquisa, 100% das participantes aprenderam sobre menstruação na escola. Essa diferença significativa pode estar relacionada ao fato da adesão da escola estudada ao Programa Dignidade Íntima^(25)^, o qual previa, além da distribuição de absorventes, a abertura para discussão de um espaço mais acolhedor, especialmente para as meninas e adolescentes cadastradas no programa. É possível que a implementação deste programa tenha proporcionado um ambiente mais favorável para a discussão e a educação sobre saúde menstrual, resultando em um aumento da conscientização e do aprendizado sobre o tema entre as participantes desta pesquisa. Assim, a presença de programas de distribuição de itens de manejo menstrual, como absorventes, em conjunto com outras ações, parece ser um fator fundamental para abordar a menstruação nas escolas, ajudando a construir uma cultura de diálogo e informação sobre os cuidados menstruais^(26)^.

Embora 11,76% das estudantes da pesquisa afirmaram possuir dificuldades para comprar absorventes, essa não é a realidade do Brasil quando comparamos com outras pesquisas. Segundo a UNICEF^(8)^, cerca de 4 milhões de meninas não têm acesso a itens básicos de cuidados menstruais, entre eles, absorventes. Dessas, quase 200 mil alunas estão privadas de condições mínimas para cuidar da sua menstruação na escola^(8)^.

No cenário internacional, essa disparidade é ainda mais acentuada. Em Bangladesh, por exemplo, mais de 80% das mulheres e meninas menstruadas utilizam materiais inadequados, como panos velhos, em vez de produtos higiênicos apropriados^(27)^, enquanto que em um estudo realizado na Etiópia, apenas 35,38% das alunas utilizavam absorventes higiênicos, e entre as alunas que não utilizaram absorventes descartáveis, 91,84% afirmaram que reutilizaram panos^(28)^. Embora a maioria das discussões acerca da pobreza menstrual estejam centradas em meninas de países de baixa e média renda, a pobreza menstrual também afeta meninas de baixa renda em países desenvolvidos, como revelou um estudo realizado no Missouri, Estados Unidos da América, em que 64% das participantes afirmaram que não puderam comprar produtos menstruais no ano anterior^(29)^.

Os resultados do presente estudo diferem do cenário geral, possivelmente, porque a escola estudada aderiu de 2021 a 2024, o Programa de Dignidade Íntima^(25)^, implantado pela Secretaria da Educação do Estado de São Paulo (Seduc-SP), que previa a disponibilidade de itens de manejo menstrual nas escolas, assim como também promovia a formação dos profissionais da escola e estudantes a respeito da pobreza menstrual.

Nesse mesmo sentido, com a crescente importância da temática e sua relevância social e equitativa, recentemente, o Ministério da Educação (MEC) iniciou uma campanha de orientação nas escolas públicas de todo o País sobre o “Programa Dignidade Menstrual: um ciclo de respeito”^(30)^, entre as iniciativas do programa, estão o encaminhamento de cartilhas, folders e vídeos informativos sobre como adquirir os absorventes gratuitos estão disponíveis pelo Programa Farmácia Popular e podem ser retirados por pessoas que tenham entre 10 e 49 anos (idade considerada fértil) e estejam inscritas no Cadastro Único para Programas Sociais (CadÚnico) do governo federal.

Embora as políticas públicas de distribuição de absorventes sejam fundamentais para combater a pobreza menstrual, a saúde menstrual vai muito além do simples acesso a esses itens. Trata-se de uma questão que envolve dignidade, bem-estar e equidade de gênero. Nesse sentido, não se pode limitar a saúde menstrual apenas aos aspectos físicos desse período. Conforme a definição desenvolvida pelo Terminology Action Group do Global Menstrual Collective, a saúde menstrual representa um estado de completo bem-estar físico, mental e social, indo além da ausência de doenças ou enfermidades relacionadas ao ciclo menstrual^(31)^.

Dentro desse conceito abrangente, o próprio Global Menstrual Collective ressalta a importância de garantir o acesso a informações seguras sobre o ciclo menstrual, adequadas para cada faixa etária, além de promover práticas de autocuidado e higiene que respeitem as preferências pessoais, garantindo conforto, privacidade e segurança. O coletivo também enfatiza a necessidade de oferecer diagnósticos e tratamentos oportunos para desconfortos e distúrbios relacionados ao ciclo menstrual, incluindo o alívio da dor. Além disso, destaca-se a importância de assegurar um ambiente acolhedor, livre de julgamentos, discriminação ou violência relacionadas à menstruação, permitindo que a pessoa que menstrua decida, sem restrições ou coerção, participar plenamente das esferas civil, cultural, econômica, social e política em todas as fases do seu ciclo menstrual^(32)^.

Entender o ciclo menstrual como uma questão de gênero, é primordial, pois pessoas do sexo masculino, que não experienciam o ciclo menstrual, são afetados pelos aspectos sociais, culturais e econômicos da menstruação^(33)^. Uma pesquisa que buscou entender a influência do gênero na representação social da menstruação, identificou que entre os jovens, homens tendem a se referir à menstruação como “suja” e que ela faz com que as mulheres sejam mais frágeis e suscetíveis às emoções^(34)^.

No ambiente escolar, essa questão é ainda mais agravada. A falta de conhecimento dos meninos sobre o ciclo menstrual frequentemente resulta em bullying e comentários depreciativos, o que intensifica a sensação de vergonha das meninas. Segundo uma pesquisa realizada em ambiente escolar, 13% das meninas já passaram por provocações menstruais e mais de 80% temem ser provocadas, especialmente por colegas do sexo masculino. As meninas lidam reduzindo a frequência escolar, a participação e a concentração na sala de aula durante os períodos^(35)^.

Diante desse cenário, é fundamental incluir a perspectiva masculina nas questões relacionadas à menstruação para promover mudanças eficazes. A educação sobre o ciclo menstrual deve começar ainda na escola, abordando tanto meninos quanto meninas. Pessoas que não menstruam expressam interesse em aprender sobre o tema^(36)^, e incluir essa questão como parte da educação sexual e das habilidades para a vida não deve ser apenas uma formalidade, mas sim uma ação concreta. Ao educar meninos sobre a gestão menstrual, eles crescerão mais informados e aptos a contribuir para a redução do estigma menstrual, criando ambientes escolares mais inclusivos e saudáveis para todos.

As principais fontes de informação sobre a menarca para as adolescentes são, em sua maioria, suas mães (39,7%), seguidas por amigas (26,6%) e professoras (21,8%)^(26)^. As atitudes e crenças que as famílias têm em relação à menstruação influenciam significativamente como meninas e meninos compreendem e lidam com o ciclo menstrual. Um estudo revelou que a probabilidade de uma boa prática de gestão da higiene menstrual foi maior entre aquelas que tinham conhecimento sobre higiene menstrual antes da menarca e tinham discutido sobre o ciclo menstrual com seus pais^(36)^.

Contudo, muitas meninas relataram que suas famílias, especialmente suas mães ou pais, não estavam abertos a discutir esses assuntos, transformando a menstruação em um tabu dentro de casa. Essa falta de diálogo franco sobre tópicos como a variação do fluxo menstrual, itens de manejo menstrual e sintomas associados, impactou negativamente a experiência menstrual dessas meninas, especialmente no início da adolescência, quando elas ainda estão se ajustando à nova realidade corporal^(36)^.

Nesse contexto, embora a dignidade menstrual tenha ganhado mais atenção e gerado políticas públicas, como as mencionadas, estudos revelam que ainda há lacunas a serem preenchidas. É fundamental considerar a experiência de outros corpos que menstruam, além de meninas e mulheres cisgênero^(37)^, bem como uma perspectiva interseccional da temática, pois a pobreza menstrual afeta mais as pessoas moradoras de periferia e negras^(38)^. Além disso, há uma necessidade urgente de mais pesquisas no Brasil que abordem adolescentes em idade escolar, assim como que explorem diversas regiões e realidades culturais do país, permitindo uma perspectiva mais abrangente e equitativa sobre o tema. Estudos futuros também serão essenciais para analisar o impacto do Programa Dignidade Menstrual, que foi implementado recentemente, possibilitando seu aprimoramento.

Como visto nesta pesquisa, persiste uma lacuna significativa no acolhimento oferecido por essas políticas de distribuição de itens de manejo menstrual, especialmente quando se trata de reconhecer e abordar a dor menstrual que afeta tantas pessoas que menstruam. Essa realidade cotidiana frequentemente é subestimada e banalizada, com as dificuldades associadas sendo naturalizadas em vez de adequadamente consideradas e enfrentadas^(39,40)^.

Como limitações deste estudo, pode-se apontar a não obtenção de um número maior de respostas ao questionário e subsequente participação na roda de conversa, o que afetou o poder de inferência na análise quantitativa. Além disso, uma das lacunas da pesquisa foi não ter envolvido os adolescentes do sexo masculino nos grupos focais, o que poderia ter contribuído para um entendimento mais amplo e inclusivo sobre a menstruação, desafiando estigmas e promovendo uma maior conscientização entre todos os gêneros. Essas limitações reforçam a necessidade de estudos futuros que ampliem o escopo de participantes e que abordam as experiências de diferentes identidades de gênero para uma visão mais completa e inclusiva.

## CONCLUSÃO

A presente pesquisa destacou como o ciclo menstrual afeta diretamente e indiretamente o cotidiano, a vida escolar e emocional das adolescentes, gerando insegurança, absenteísmo escolar e possível dificuldade no acesso a produtos de manejo menstrual. Entre os principais resultados, coloca-se que mais da metade das adolescentes já deixou de ir à escola quando menstruada e relataram se sentirem inseguras durante este período. Apesar da escola ser uma fonte central de aprendizado sobre o tema, questões como vergonha e estigmatização ainda persistem, especialmente proveniente dos adolescentes do sexo masculino. Ao contar experiências das adolescentes, o estudo contribui para a visibilidade do ciclo menstrual como uma temática importante, apontando para a necessidade de intervenções educativas e familiares que ampliem o diálogo e o apoio, promovendo maior acolhimento e compreensão dos desafios menstruais. Esses achados reforçam a necessidade de ações intersetoriais que integrem educação, saúde e assistência social para garantir dignidade menstrual, combater estigmas e promover ambientes escolares mais inclusivos e equitativos.

## DISPONIBILIDADE DE DADOS

Todo o conjunto de dados que dá suporte aos resultados deste estudo está disponível mediante solicitação ao autor correspondente.

# Translation: Salud menstrual: análisis de las experiencias de adolescentes en edad escolar

**DOI:** 10.1590/1980-220X-REEUSP-2025-0003es

**Authors:** Jennifer Caroline de Oliveira Adomaitis, Natália Sevilha Stofel, Maria Gabriele Formis Silva, Vivian Campos Santos, Flávio Adriano Borges, Diene Monique Carlos, Bruna Luana de Souza Farias, Márcia Niituma Ogata, Juliana Cristina dos Santos Monteiro

**Affiliations:** 1 Universidade Federal de São Carlos, Departamento de Enfermagem, São Carlos, SP, Brasil.; 2 Universidade de São Paulo, Escola de Enfermagem de Ribeirão Preto, Ribeirão Preto, SP, Brasil.; 3 Universidade Federal de São Carlos, Programa de Pós-Graduação em Enfermagem, São Carlos, SP, Brasil.

**Keywords:** Adolescente, Menstruación, Instituciones Académicas

## Abstract

**Objetivo:**

Comprender las percepciones de las adolescentes en edad escolar sobre el ciclo menstrual.

**Método:**

Se trata de un estudio exploratorio con métodos mixtos realizado en una escuela técnica estatal del interior de São Paulo. Se realizó un análisis estadístico descriptivo de los datos cuantitativos y se utilizó el software Iramuteq para los datos cualitativos. Los resultados se compararon con los principios de la justicia reproductiva.

**Resultados:**

Participaron en este estudio 34 adolescentes. Se observó que el 17% tenía dificultades para comprar toallas sanitarias, el 88% se sentía inseguro durante la menstruación y el 53% había faltado a la escuela por estar menstruando. Todas afirmaron que habían aprendido sobre la menstruación en la escuela. Surgieron cinco categorías temáticas: 1) Retos de la menstruación en la escuela; 2) Educación y conversaciones sobre la menstruación en el entorno familiar; 3) Apoyo y asesoramiento; 4) Dificultades físicas y prácticas de la menstruación; 5) Vergüenza y estigmatización.

**Conclusión:**

La investigación revela que la menstruación tiene un impacto significativo en la vida de las adolescentes, causando inseguridad y absentismo. Las escuelas desempeñan un papel importante en la difusión de información sobre el tema, y los datos obtenidos apuntan a la necesidad de ampliar el debate y reducir el estigma.

## INTRODUCCIÓN

La menstruación, aunque es un proceso fisiológico, suele ir acompañada no solo de molestias físicas y cambios emocionales, sino también de estigmas, por lo que se considera un tabú. En diversas culturas, existe una tendencia a asociar la menstruación con algo impuro o negativo^(1)^. El patriarcado, como normalizador de los cuerpos y los comportamientos sociales, al incidir en el ciclo menstrual, produce y reproduce desinformación, creando miradas peyorativas y situando a la menstruación en un lugar que despierta repugnancia^(2)^. Tener esta visión asociada al cuerpo biológicamente femenino segrega aún más a las personas que menstrúan^(3)^.

La pobreza menstrual se refiere a la dificultad o falta de acceso a productos esenciales para el manejo de la menstruación, así como a la falta de lugares adecuados para utilizarlos. Esto incluye la ausencia de servicios básicos de saneamiento y la limitación en el acceso a información sobre la menstruación^(4)^. En esencia, la pobreza menstrual es un grave problema social que refleja las desigualdades sociales y de género, especialmente si comparamos el alto costo de los productos para el manejo de la menstruación con otros artículos de cuidado personal^(5)^. La menstruación se invisibiliza, lo que hace que no se discuta, y los problemas no se observan, sin embargo, no desaparecen y afectan intensamente a las personas más vulnerables^(6)^.

Además de la dificultad de acceso a los productos y la falta de infraestructura, es importante considerar que la menstruación implica una serie de necesidades relacionadas con la salud física, emocional y social. El concepto de salud menstrual se define como un estado de bienestar físico, mental y social en relación con el ciclo menstrual, y no solo la ausencia de enfermedades o dolencias. Este enfoque amplía la mirada más allá de las condiciones materiales, incluyendo también el derecho a la información sobre la menarquia, el acceso a espacios seguros y privados para cuidar la higiene menstrual y la posibilidad de participar plenamente en actividades educativas, sociales y profesionales durante el período menstrual^(7)^.

Reconocer la salud menstrual como un derecho fundamental implica considerar que tanto la pobreza menstrual como la falta de cuidados adecuados para la salud menstrual contribuyen a impactos negativos en la vida de las personas que menstrúan, entre los impactos generados, uno de los más significativos es el absentismo escolar. Según datos de un estudio de lo Fondo de Población de las Naciones Unidas, una de cada diez niñas falta a la escuela cuando está menstruando^(8)^. En Brasil, según datos de la guía de implementación del Programa de Dignidad Menstrual, una de cada cuatro niñas falta a la escuela cuando está menstruando porque no puede comprar absorbentes; alrededor de 4 millones de niñas sufren al menos una privación de recursos en las escuelas (acceso a absorbentes y/o instalaciones básicas, como baños y jabones); además, solo el 20% de las alumnas se sentían bien informadas cuando tuvieron su primera menstruación^(9)^.

La falta de información y de acceso a productos para el manejo de la menstruación, junto con los prejuicios y las dificultades, genera incomodidad, vergüenza e incluso acoso, lo que da lugar a la exclusión de las niñas de diversas actividades cotidianas y repercute negativamente en su rendimiento escolar y profesional, en comparación con las personas que no menstrúan^(10)^. Además, los estigmas hacen que las niñas eviten cambiarse la compresa en la escuela, lo que compromete su salud física. La «etiqueta menstrual» también refuerza la idea de que no se debe hablar abiertamente sobre la menstruación, lo que perjudica la autoestima y la confianza de las niñas. Este estigma se basa en la concepción de que la sangre menstrual es sucia o impura, algo que debe evitarse^(11)^.

Otros factores que contribuyen al absentismo son los síntomas físicos asociados a la menstruación, como la dismenorrea, que se caracteriza por intensos dolores menstruales de origen uterino. Uno de los estudios indica que esta afección afecta hasta al 95% de las mujeres que menstrúan^(12)^; el síndrome premenstrual, caracterizado por un conjunto de manifestaciones físicas, emocionales y conductuales que surgen durante la fase lútea del ciclo menstrual (generalmente en la semana anterior a la menstruación) y tienden a desaparecer con el inicio del sangrado menstrual^(13)^.

Además de los síntomas físicos, la menstruación también genera impactos emocionales significativos. Un estudio transversal realizado en Japón en 2022 con 198 estudiantes de secundaria reveló que los síntomas menstruales, como el dolor intenso (dismenorrea), el impacto en las actividades cotidianas, la menstruación inesperada y la necesidad frecuente de tomar analgésicos, estaban fuertemente asociados con puntuaciones más bajas en la calidad de vida, especialmente en los aspectos relacionados con la salud mental^(14)^. Los impactos de estos síntomas son profundos y pueden comprometer la calidad de vida, interfiriendo en las actividades diarias y conduciendo a la exclusión escolar, profesional y social. A pesar de su alta prevalencia, muchas personas se enfrentan a un apoyo sanitario insuficiente o inhumano^(15)^.

La realidad de que alrededor de 800 millones de personas menstrúan cada día en todo el mundo revela la magnitud del problema, poniendo de manifiesto la necesidad de un sistema de políticas públicas que garantice el acceso a productos de higiene, una educación adecuada sobre la salud menstrual y una infraestructura de saneamiento y agua^(16)^. Además, el estigma y los tabúes asociados a la menstruación, así como la falta de un manejo adecuado de los síntomas del periodo, son cuestiones que solo pueden abordarse mediante políticas que promuevan la igualdad de género, la inclusión y el respeto de los derechos humanos^(17)^.

Así, ante la escasez de abordajes sobre el tema y los impactos que genera, el presente trabajo tuvo como objetivo comprender las percepciones de las adolescentes escolares sobre el ciclo menstrual.

## MÉTODO

### Tipo de Estudio

Se trató de una investigación de métodos mixtos, descriptiva, analítica y exploratoria, con un enfoque cuantitativo-cualitativo definido siguiendo la recomendación de la *Mixed Methods Appraisal Tool* (MMAT)^(18)^, ya que la complejidad del fenómeno estudiado requería la complementariedad de los méto-dos^(19)^. Así, primero se llevó a cabo la Recolección de datos cuantitativos y, a continuación, la Recolección de datos cualitativos.

### Lugar

El presente estudio se llevó a cabo en una Escuela Técnica Estatal (ETEC) en un municipio pequeño, en el interior del estado de São Paulo, Brasil. La ETEC ofrece cinco cursos técnicos integrados a los tres años de la educación secundaria. En 2023, el total de matriculados era de 215 estudiantes.

### Criterios de Selección y Definición de la Muestra

Los criterios de inclusión fueron: adolescentes (de 12 a 18 años cumplidos, según el Estatuto del Niño y del Adolescente) que ya hubieran pasado por la menarquia y estuvieran matriculadas regularmente en la escuela. Los criterios de exclusión fueron: adolescentes que no pudieran comprender las instrucciones o el contenido de la investigación, ya fuera por barreras lingüísticas, cognitivas o de comunicación; y adolescentes que, en el momento de la recopilación, estuvieran embarazadas. Para la selección de las adolescentes, se celebró inicialmente una reunión con la coordinación pedagógica de la ETEC, que enumeró algunos horarios y clases en los que se consideraba que la investigación era más pertinente, de acuerdo con las demandas previas que llegaban a los docentes. Las adolescentes de las clases previamente enumeradas fueron entonces invitadas a participar en una reunión celebrada en la escuela, en la que una de las investigadoras presentó el proyecto. Así, participaron en esta investigación: adolescentes que menstruaban, cuyos responsables firmaron el Término de Asentimiento y ellas firmaron el Término de Consentimiento Libre y Esclarecido. Se trató, por lo tanto, de una muestra por conveniencia que contó con 34 participantes, es decir, todas las adolescentes de las clases señaladas por la coordinación como prioritarias.

### Recopilación de Datos

El acercamiento con la ETEC fue realizado por la profesora orientadora que coordinaba el proyecto, directamente con la coordinadora pedagógica de la escuela, que también era la responsable del programa «Dignidad Íntima» en la ETEC. El programa «Dignidad Íntima» era una iniciativa del gobierno del estado de São Paulo, que reservaba una partida presupuestaria para la compra y distribución de absorbentes menstruales en las escuelas estatales.

La recolección de datos fue realizada por dos investigadoras previamente capacitadas en un grupo de investigación, donde recibieron formación sobre la metodología de las rondas de conversación y aspectos específicos de la investigación con adolescentes. La recolección de datos se realizó en dos etapas: la primera se caracterizó por un enfoque cuantitativo, mediante el autollenado de un cuestionario de caracterización, elaborado a partir de la combinación del cuestionario de la Pesquisa Nacional de Saúde do Escolar (PeNSE) de 2015^(20)^ y el cuestionario de diversidad e inclusión de 2018^(21)^.

En la segunda etapa de enfoque cualitativo, las investigadoras capacitadas, acompañadas por la coordinadora del proyecto en calidad de observadora, condujeron grupos focales^(22)^ con un guion semiestructurado (Cuadro 1) con las adolescentes que participaron en la primera etapa y aceptaron continuar. Las reuniones se llevaron a cabo de manera presencial en la biblioteca de la ETEC, en el intervalo entre el turno matutino y vespertino. Se realizaron tres grupos focales entre los meses de agosto y septiembre de 2023, con una duración media de una hora. El primero tuvo lugar el 18 de agosto de 2023, con estudiantes de primer año de secundaria integrados en el curso técnico de Administración. Los dos siguientes se celebraron el 1 de septiembre de 2023: uno con alumnas de segundo año de secundaria integradas en el curso técnico de Marketing y otro con estudiantes de tercer año de secundaria integradas en el curso técnico de Administración.

**Cuadro 1:** Guión semiestructurado – São Carlos, SP, Brasil, 2023.

1.¿A qué edad tuviste tu primera menstruación y cómo fue?
2. ¿Qué sabes sobre la menstruación?
3. ¿Utilizas algún tipo de absorbente o copa menstrual? ¿Cómo fue esa elección?
4. ¿Quién compra tus absorbentes? ¿Tienes acceso a ellos todos los meses?
5. ¿Alguna vez te has sentido inseguro/a por estar menstruando? Cuéntanos un poco más sobre eso, por favor.
6. ¿Alguna vez has faltado a la escuela por estar menstruando? ¿Cuál fue el motivo?
7. ¿Conoces a alguien que tenga dificultades para comprar absorbentes?
8. ¿Has aprendido sobre la menstruación en tu escuela? ¿Cómo se abordó el tema?
9. ¿Cómo contribuye tu escuela a promover la dignidad menstrual?
10. ¿Cómo crees que debería promoverse la dignidad menstrual en la escuela?

Fuente: Autores.

### Análisis de los Datos

Los cuestionarios auto-completados fueron recopilados y digitados en hojas de cálculo del software Excel^®^. El programa estadístico utilizado fue Stata, versión 12.0, y se realizó un análisis descriptivo para caracterizar la muestra. Todo el material de las rondas de conversación fue grabado y transcrito. Se compuso un corpus textual mediante la unión de las respuestas presentadas, siguiendo con su procesamiento y un primer análisis mediante el software Interface de R pour les Analyses Multidimensionnelles de Textes et de Questionnaires (IRAMUTEQ^®^). Así, las respuestas a las preguntas abiertas se organizaron para componer el corpus textual, que se preparó y revisó con el fin de eliminar errores tipográficos y estandarizar siglas y expresiones (conservando los mismos significados). El método organiza las formas léxicas en clases, atribuyendo una importancia relativa a cada una de ellas. Las ocurrencias de cada una de las clases se consideraron a partir de valores estadísticamente significativos (p < 0,05).

Los resultados obtenidos se leyeron y discutieron a través de los principios de la Justicia Reproductiva. La Justicia Reproductiva aportó al paradigma de los derechos sexuales y reproductivos cuestiones planteadas por el decolonialismo y la interseccionalidad, es decir, una perspectiva crítica para contrarrestar los patrones sexistas, racistas, entre otros marcadores sociales de desigualdad^(23)^.

### Aspectos Éticos

El estudio fue aprobado por el Comité de Ética en Investigación de la Universidad Federal de São Carlos, con el número de dictamen 6.308.853. Todas las participantes firmaron el Término de Consentimiento Libre y Esclarecido y sus responsables, el Término de Asentimiento.

## RESULTADOS

En total, 34 estudiantes participaron en la investigación. En la Tabla 1 se presenta el perfil de las respuestas obtenidas. La mayoría se declaró mestiza (47,06%), mujer cisgénero (85%) y heterosexual (61,76%). De las 34 estudiantes, se constató que el 11.76% (4) de las participantes tienen dificultades para comprar absorbentes, el 88.24% (30) se sienten inseguras cuando están menstruadas, mientras que el 52.94% (18) han dejado de ir a la escuela por estar menstruadas. Todas las entrevistadas afirmaron haber aprendido sobre la menstruación en la escuela.

**Tabla 1:** Caracterización de las adolescentes entrevistadas – São Carlos, SP, Brasil, 2023.

Variable	Proporción (n)
**Raza**	
Parda	47,06% (16)
Negra	11,76% (4)
Blanca	41,18% (14)
**Edad**	
15 años	17,65% (6)
16/17 años	70,59% (24)
18 años	8,82% (3)
Prefiero no responder	2,94% (1)
**Género**	
Mujer Cis	85,0% (29)
Prefiero no responder	15,0% (5)
**Orientación Sexual**	
Heterosexual	61,76% (21)
Bisexual	20,59% (7)
Lésbica	2,94% (1)
Demisexual	2,94% (1)
Prefiero no responder	11,77 (4)
**¿Tiene dificultades para comprar absorbentes?**	
Si	11,76% (4)
No	88,24% (30)
**¿Se siente insegura cuando está menstruando?**	
Si	88,24% (30)
No	8,82% (3)
No respondió	2,94% (1)
**¿Dejó alguna vez de ir a la escuela en el período menstrual?**	
Si	52,94% (18)
No	47,06% (16)
**¿Aprendió sobre la menstruación en la escuela?**	
Si	100,0% (34)

Fuente: Autores.

En lo que respecta al análisis cualitativo de los datos de la investigación, el corpus general estuvo compuesto por tres textos, que corresponden a las transcripciones de las rondas de conversación, separadas en 106 segmentos de texto (st), con un aprovechamiento de 66 st (62,26%) y 3552 ocurrencias (palabras, formas o vocabulario). El contenido analizado se clasificó en cinco categorías: Categoría 1 – Retos de la menstruación en la escuela – con 13 st (19,7%), Categoría 2 – Educación y conversaciones sobre la menstruación en el entorno familiar – con 12 st (18,18%), Categoría 3 – Apoyo y asesoramiento – con 12 st (18,18%), Clase 4 – Dificultades físicas y prácticas de la menstruación – con 18 st (27,27%) y Clase 5 – Vergüenza y estigmatización – con 11 st (16,67%).

Cabe destacar que estas 5 clases se dividen en ramificaciones, siendo las clases con ramificaciones más cercanas las que presentan mayor similitud (clase 3 y clase 5), mientras que las ramificaciones superiores (clase 2 y clase 4) son más distintas de las demás (Figura 1).

**Figura 1:** Dendograma de análisis del corpus textual de las rondas de conversación.

Fuente: Autores.

En este sentido, tenemos el primer tema denominado «Desafíos y estigma relacionados con la menstruación», que abarca las clases 1 (Desafíos de la menstruación en la escuela), 5 (Vergüenza y estigmatización) y 3 (Apoyo y asesoramiento). El segundo tema es «Dificultades físicas y prácticas de la menstruación», representado por la clase 4 (Dificultades físicas y prácticas de la menstruación) y, por último, el tercer tema es «Aprendizajes y conversaciones sobre la menstruación en el entorno familiar», representado por la clase 2 (Aprendizajes y conversaciones sobre la menstruación en el entorno familiar). A continuación se describen y ejemplifican cada una de estas clases.

### Clase 1: Desafíos de la Menstruación en la Escuela

Comprende el 19,7% (13 st) del corpus total analizado. Constituida por palabras y radicales en el intervalo entre x^2^ = 2,47 (período) y x^2^ = 22,23 (niño), esta clase está compuesta por palabras como «escuela» (x^2^ = 14,54); «salir» (x^2^ = 8,23); «conversación» (x^2^ = 4,38) y «proyecto» (x^2^ = 4,38).

A partir del análisis, se puede verificar que menstruar en el entorno escolar puede ser incómodo y estigmatizante, especialmente cuando este tema no se aborda con los niños, que a menudo reproducen prejuicios al respecto, como se puede observar en los siguientes fragmentos:

*Los chicos no tienen ni idea. Se burlan, se ríen y piensan que la chica está manchada porque quiere. Es sangre, pero ellos reaccionan con asco, diciendo que es sucio. Y nosotras no podemos controlar eso (2 MKT).*

*A veces, esto es complicado porque la mujer siente que el hombre se avergonzará de ella. Y esta sigue siendo una percepción que persiste entre algunos chicos, en cierto modo (3 ADM).*

*Hubo una situación en la que una niña menstruó sin saberlo y la sangre terminó manchándola. Los chicos comenzaron a burlarse de ella y ella se fue muy avergonzada. Siguieron recordándoselo y burlándose de ella durante aproximadamente un mes. Fue algo muy desagradable, pero ellos no tenían mucho conocimiento sobre el tema (3 ADM).*

Los relatos señalan a la escuela como un espacio donde la experiencia de la menstruación puede estar marcada por la vergüenza, el miedo a la exposición y el sufrimiento psíquico. El análisis demuestra que el silencio en torno al tema, especialmente en relación con los chicos, refuerza la desinformación y fomenta actitudes discriminatorias. La ausencia de un enfoque educativo universal sobre la salud menstrual contribuye a un ambiente escolar excluyente, donde la falta de empatía y el acoso comprometen el bienestar físico y emocional de las estudiantes que menstrúan. En este sentido, la escuela, como espacio formativo, debe asumir un papel activo en la deconstrucción de los estigmas y la promoción de la equidad de género.

### Clase 2 – Aprendizajes y Conversaciones Sobre la Menstruación en el Entorno Familiar

Comprende el 18,18% (12 st) del corpus total analizado. Constituida por palabras y radicales en el intervalo entre x^2^ = 2,14 (yo) y x^2^ = 40,83 (madre), esta clase está compuesta por palabras como «explicar» (x^2^ = 24,34); «hablar» (x^2^ = 5,82); «menstruación» (x^2^ = 5,3); «hermano» (x^2^ = 4,97).

A través del análisis, se percibe la importancia de la educación menstrual dentro del entorno familiar, especialmente para desmitificar tabúes, promover la salud y el bienestar, y crear un espacio seguro para discutir cuestiones relacionadas con el ciclo menstrual. Además, hablar con las niñas que aún no han menstruado es importante para prepararlas para la pubertad, ayudándolas a desmitificar el proceso y reducir la ansiedad, así como a manejar la menstruación de forma segura, como se puede observar en los fragmentos de texto:

*Me avergoncé cuando mi abuelo llegó del trabajo y mi abuela lo sentó a la mesa y dijo a todos: «¿Ya saben quién se ha hecho mayor?». Yo tenía 11 años y me sentí muy incómoda. Todos se quedaron mirándome y yo me quedé sentada, sintiéndome muy avergonzada (2 MKT).*

*Mi mamá cree que la menstruación es un gran tabú en casa. Por ejemplo, cuando menstrué, no me explicó nada; solo me dijo que usara un tampón y tomara un medicamento (3 ADM).*

*Menstrué a los 12 años y no sabía nada sobre el tema. Quien me enseñó a ponerme un tampón fue mi prima, que tenía 16 años (3 ADM).*

*Mi mamá siempre nos ha hablado de estos temas desde que éramos pequeñas. Nos explicó que, cuando era joven, su abuela no hablaba de eso y, hasta hoy, no le gusta que lo hablemos. Por eso, mi mamá cambió esa realidad y nos hablaba desde pequeñas. Así, cuando me vino la regla, ya sabía bastante sobre el tema (3 ADM).*

*Mi mamá no solía explicarme mucho, pero me fui a vivir con mi abuela por un tiempo y estuve alejada de ella. Fue en ese periodo cuando me vino la regla y me sentí desesperada, porque no sabía lo que estaba pasando (1 ADM).*

*Creo que, a menudo, falta comprensión por parte de la familia (3 ADM).*

Las declaraciones evidencian que la familia desempeña un papel central en la construcción de las percepciones sobre la menstruación y en la preparación emocional y práctica para vivirla, y que, cuando el tema se trata con naturalidad y franqueza, se reduce la ansiedad y el miedo y se gana seguridad en el manejo del ciclo. Sin embargo, muchos relatos revelan que el entorno familiar sigue estando marcado por silencios, tabúes y vergüenzas, lo que refuerza los sentimientos de vergüenza e inseguridad en las niñas. Esta falta de diálogo, a menudo arraigada en patrones culturales que tratan la menstruación como algo prohibido o sucio, compromete el acceso a información esencial para el cuidado y el bienestar. Ante esto, se hace urgente invertir en políticas públicas que incluyan acciones de educación en salud dirigidas a las familias, abordando la menstruación de manera integral, afectiva e intergeneracional, con el fin de romper los ciclos de desinformación y promover una cultura de cuidado, dignidad y respeto desde los primeros signos de la pubertad.

### Clase 3 – Apoyo y Asesoramiento

Comprende el 18,18% (12 st) del corpus total analizado. Constituida por palabras y radicales en el intervalo entre x^2^ = 2,42 (menstruación) y x^2^ = 14,14 (ayudar), esta clase está compuesta por palabras como «charla» (x^2^ = 9,24) y «falta» (x^2^ = 4,97).

El apoyo y el asesoramiento son fundamentales para lidiar con el ciclo menstrual, especialmente en contextos donde el acceso a productos menstruales es limitado. Muchas niñas y mujeres enfrentan no solo desafíos físicos, como cólicos y cambios de humor, sino también la presión social y la falta de información adecuada sobre su ciclo. En este sentido, la escuela tiene un papel fundamental para reducir estos desafíos, mediante la disponibilidad de productos para el manejo menstrual, como los absorbentes. Además, debe brindar orientación sobre su uso y cuidados para garantizar que las mujeres no se sientan aisladas o avergonzadas durante este período, reduciendo incluso el absentismo escolar y los prejuicios, como se puede ver a continuación:

*Esto es muy reconfortante, porque sabemos que, si lo necesitamos, habrá alguien con quien hablar y de quien recibir absorbentes aquí en la escuela (2 MKT).*

*Hubo una charla sobre la menstruación en la que también se incluyó a los chicos (2 MKT).*

*Creo que sería importante una charla que incluyera a todas las personas, no solo a las mujeres, sino también a los chicos, para que comprendan mejor cómo apoyar a las chicas durante este periodo (1 ADM).*

*Es una fase en la que estamos muy sensibles y necesitamos apoyo durante este periodo (3 ADM).*

*Hay un proyecto en el que quien quisiera o tuviera alguna dificultad firmaba un documento para recibir mensualmente absorbentes y toallitas húmedas enviadas por la secretaría (1 ADM).*

*Los absorbentes vienen en una cajita empaquetada, de manera que no se puede identificar lo que hay dentro. Ella nos mostró que eran absorbentes, porque no se puede saber lo que es. También es posible elegir la cantidad necesaria, como 8, 16 o 46 unidades, y el suministro se realiza mensualmente. El material se entrega discretamente, en un rincón, para que nadie lo sepa (1 ADM).*

El análisis de los relatos muestra la importancia del apoyo institucional para afrontar los retos asociados al ciclo menstrual, especialmente en contextos de vulnerabilidad social y económica. La escuela surge como un espacio estratégico y transformador para acoger y promover la salud menstrual, ya sea mediante la oferta de productos de higiene íntima, como absorbentes y toallitas húmedas, o mediante la realización de actividades educativas que promuevan la información, reduzcan el estigma y fomenten la equidad de género. Estos hallazgos refuerzan la necesidad de políticas públicas que articulen la salud, la educación y la asistencia social, como una tríada capaz de contribuir a la reducción de la pobreza menstrual y la permanencia escolar.

### Clase 4 – Dificultades Físicas y Prácticas de la Menstruación

Comprende el 27,27% (18 st) del corpus total analizado. Constituida por palabras y radicales en el intervalo entre x^2^ = 2,2 (poder) y x^2^ = 14,43 (dolor), esta clase está compuesta por palabras como «ropa» (x^2^ = 11,35); «acceso» (x^2^ = 11,35); «absorbentes» (x^2^ = 5,5); “baño” (x^2^ = 3,82); “incómodo” (x^2^ = 2,46).

Mediante el análisis, fue posible identificar las dificultades físicas y prácticas del ciclo menstrual, incluyendo síntomas como cólicos, fatiga y cambios de humor, que pueden afectar la rutina diaria. Además, el manejo práctico de la menstruación, como la necesidad de tener acceso a productos adecuados y un lugar privado para el cambio, puede ser un reto, especialmente en contextos donde estos artículos son escasos o donde hay falta de infraestructura. Estas dificultades pueden generar malestar físico y emocional, lo que conduce a una experiencia negativa del ciclo menstrual, además de contribuir significativamente al absentismo escolar. A continuación se presentan algunos ejemplos:

*No hay condiciones dignas en esos días, a menudo no hay absorbentes ni medicamentos para usar. Muchas mujeres no pueden comprar absorbentes, medicamentos o incluso toallitas húmedas. Sabemos que, en algunos casos, algunas tienen que usar papel. Tuvimos una conversación sobre esto, y algunas chicas llegaron a usar miga de pan (2 MKT).*

*Creo que eso es pobreza, porque no tienen condiciones dignas en esos días que ya son difíciles de afrontar, y además tienen que pasar por eso sin ningún recurso (2 MKT).*

*Sobre todo las mujeres con flujo menstrual intenso, que tienen dificultades para bañarse en contextos rurales, donde a menudo no hay acceso al agua, como ella mencionó (2 MKT).*

*Asocié el dolor, el cuidado y el regaño, porque a veces quería hacer ciertas cosas, pero no podía (2 MKT).*

*Viajé a la playa hace un mes y tenía mucho dolor debido a los intensos cólicos que tengo. Tomé medicamentos, pero me sentí mal porque no pude meterme al agua (2 MKT).*

El análisis de las declaraciones de esta clase pone de manifiesto las percepciones de las adolescentes ante las múltiples dimensiones del sufrimiento menstrual, que van más allá de los síntomas físicos como el dolor, la fatiga y los cambios de humor, e incluyen también barreras materiales y estructurales. La falta de absorbentes, medicamentos y acceso a baños adecuados hace que el ciclo menstrual sea una experiencia marcada por la incomodidad, la inseguridad y la exclusión, que se intensifica entre las niñas en situación de vulnerabilidad.

### Clase 5 – Vergüenza y Estigmatización

Comprende el 16,67% (11 st) del corpus total analizado. Constituida por palabras y radicales en el intervalo entre x^2^ = 2,12 (sentir) y x^2^ = 45,52 (vergüenza), esta clase está compuesta por palabras como «derecho» (x^2^ = 15,71) y «mujer» (x^2^ = 13,64).

Los datos muestran que el estigma en torno a la menstruación tiene un profundo impacto en la autoestima de las personas que menstrúan, lo que a menudo conduce a la vergüenza y al silencio en torno a un proceso natural. La desvalorización de la menstruación puede minar la confianza y dificultar la plena participación en actividades sociales y profesionales. Además, el estigma contribuye a la negación de derechos básicos, como el acceso a productos menstruales y a la educación sobre salud menstrual, perpetuando las desigualdades de género, como se puede ver en los siguientes extractos:

*Creo que todas las mujeres tienen derecho a tener absorbentes en casa y a no tener dificultades para conseguirlos (2 MKT).*

*Menstruar es una experiencia negativa porque genera miedo al juicio de otras personas (2 MKT).*

*Además de la vergüenza, a menudo lo que usaban no era suficiente para contener el flujo, lo que resultaba en manchas en la ropa y comentarios de las personas, lo que aumentaba la incomodidad (3 ADM).*

*Cualquier pequeño signo de sangre ya es motivo de comentarios, lo que genera inseguridad en cualquier mujer (3 ADM).*

*Las mujeres a menudo no pueden hablar de lo que sienten y viven por vergüenza y miedo a ser juzgadas (1 ADM).*

Los testimonios evidencian que la vergüenza asociada a la menstruación no es solo una experiencia individual, sino un reflejo de cómo la sociedad trata el tema como un tabú, considerándolo algo «sucio» y «vergonzoso», y que las personas que menstrúan no deben hablar sobre el tema. Este silencio afecta al bienestar emocional y limita la participación de las personas que menstrúan en diferentes ámbitos de la vida. Superar esta situación requiere no solo la distribución de insumos, sino también una transformación cultural a través de la educación y la valoración de la experiencia menstrual como parte de la salud y la dignidad humana.

## DISCUSIÓN

El presente estudio presentó las percepciones de las adolescentes en edad escolar sobre el ciclo menstrual y cómo este afecta el absentismo escolar y la confianza de las adolescentes, ya que, aunque la mayoría no tiene dificultades para adquirir artículos para el manejo de la menstruación, como absorbentes desechables, una gran parte no va a la escuela cuando menstrúa y se siente insegura en esta fase del ciclo.

En lo que respecta al absentismo escolar, en 2021, UNICEF y UNFPA^(24)^ realizaron una encuesta sobre salud y dignidad menstrual, a través de la plataforma U-Report, en todo Brasil, en la que respondieron voluntariamente 1730 personas, la mayoría de entre 13 y 24 años. Entre ellas, el 62% afirma que ha dejado de ir a la escuela u otros lugares debido a la menstruación. Además, el 73% dijo que se ha sentido avergonzada en la escuela o en otro lugar público debido a la menstruación. Esto corrobora los datos de la presente investigación, ya que el 52,94% de las estudiantes dejaron de ir a la escuela mientras menstruaban y el 88,24% se sintieron inseguras durante ese período.

En esa misma investigación^(24)^, el 71% de las personas que menstruan dijeron que nunca habían tenido clases, charlas o círculos de conversación sobre cuidados durante la menstruación en la escuela. Mientras que en esta investigación, el 100% de las participantes aprendieron sobre la menstruación en la escuela. Esta diferencia significativa puede estar relacionada con el hecho de que la escuela estudiada se adhirió al Programa Dignidad Íntima^(25)^, que preveía, además de la distribución de absorbentes, la apertura a la discusión de un espacio más acogedor, especialmente para las niñas y adolescentes inscritas en el programa. Es posible que la implementación de este programa haya proporcionado un ambiente más favorable para el debate y la educación sobre la salud menstrual, lo que ha dado lugar a un aumento de la concienciación y el aprendizaje sobre el tema entre las participantes en esta investigación. Así, la presencia de programas de distribución de artículos para el manejo menstrual, como los absorbentes, junto con otras acciones, parece ser un factor fundamental para abordar la menstruación en las escuelas, ayudando a construir una cultura de diálogo e información sobre los cuidados menstruales^(26)^.

Aunque el 11,76% de las estudiantes de la investigación afirmaron tener dificultades para comprar absorbentes, esta no es la realidad de Brasil cuando la comparamos con otras investigaciones. Según UNICEF^(8)^, alrededor de 4 millones de niñas no tienen acceso a artículos básicos para el cuidado menstrual, entre ellos, absorbentes. De ellas, casi 200 000 alumnas carecen de las condiciones mínimas para cuidar de su menstruación en la escuela^(8)^.

En el panorama internacional, esta disparidad es aún más acusada. En Bangladesh, por ejemplo, más del 80% de las mujeres y niñas menstruantes utilizan materiales inadecuados, como trapos viejos, en lugar de productos higiénicos apropiados^(27)^, mientras que en un estudio realizado en Etiopía, solo el 35.38% de las alumnas utilizaban absorbentes higiénicos, y entre las alumnas que no utilizaban absorbentes desechables, el 91.84% afirmaron que reutilizaban trapos^(28)^. Aunque la mayoría de los debates sobre la pobreza menstrual se centran en las niñas de países de ingresos bajos y medios, la pobreza menstrual también afecta a las niñas de bajos ingresos en países desarrollados, como reveló un estudio realizado en Missouri, Estados Unidos, en el que el 64% de las participantes afirmaron que no pudieron comprar productos menstruales durante el año anterior^(29)^.

Los resultados del presente estudio difieren del panorama general, posiblemente porque la escuela estudiada se adhirió de 2021 a 2024 al Programa de Dignidad Íntima^(25)^, implantado por la Secretaría de Educación del Estado de SP, que preveía la disponibilidad de artículos para el manejo menstrual en las escuelas, así como la formación de los profesionales de la escuela y los estudiantes sobre la pobreza menstrual.

En este mismo sentido, dada la creciente importancia del tema y su relevancia social y equitativa, recientemente, el Ministerio de Educación (MEC) inició una campaña de orientación en las escuelas públicas de todo el país sobre el «Programa Dignidad Menstrual: un ciclo de respeto»^(30)^. Entre las iniciativas del programa se encuentran el envío de folletos, carpetas y videos informativos sobre cómo adquirir los absorbentes gratuitos que están disponibles a través del Programa Farmacia Popular y que pueden ser retirados por personas de entre 10 y 49 años (edad considerada fértil) y que estén inscritas en el Registro Único para Programas Sociales (CadÚnico) del gobierno federal.

Aunque las políticas públicas de distribución de absorbentes son fundamentales para combatir la pobreza menstrual, la salud menstrual va mucho más allá del simple acceso a estos artículos. Se trata de una cuestión que implica dignidad, bienestar y equidad de género. En este sentido, no se puede limitar la salud menstrual solo a los aspectos físicos de este período. Según la definición elaborada por el Grupo de Acción Terminológica del Colectivo Menstrual Global, la salud menstrual representa un estado de completo bienestar físico, mental y social, que va más allá de la ausencia de enfermedades o dolencias relacionadas con el ciclo menstrual^(31)^.

Dentro de este concepto amplio, el propio Colectivo Menstrual Global destaca la importancia de garantizar el acceso a información segura sobre el ciclo menstrual, adecuada para cada grupo de edad, además de promover prácticas de autocuidado e higiene que respeten las preferencias personales, garantizando la comodidad, la privacidad y la seguridad. El colectivo también enfatiza la necesidad de ofrecer diagnósticos y tratamientos oportunos para las molestias y trastornos relacionados con el ciclo menstrual, incluido el alivio del dolor. Además, se destaca la importancia de garantizar un entorno acogedor, libre de juicios, discriminación o violencia relacionados con la menstruación, que permita a la persona que menstrúa decidir, sin restricciones ni coacción, participar plenamente en las esferas civil, cultural, económica, social y política en todas las fases de su ciclo menstrual^(32)^.

Entender el ciclo menstrual como una cuestión de género es fundamental, ya que las personas de sexo masculino, que no experimentan el ciclo menstrual, se ven afectadas por los aspectos sociales, culturales y económicos de la menstruación^(33)^. Una investigación que buscaba comprender la influencia del género en la representación social de la menstruación identificó que, entre los jóvenes, los hombres tienden a referirse a la menstruación como «sucia» y que hace que las mujeres sean más frágiles y susceptibles a las emociones^(34)^.

En el entorno escolar, esta cuestión se agrava aún más. La falta de conocimiento de los niños sobre el ciclo menstrual a menudo da lugar a acoso y comentarios despectivos, lo que intensifica la sensación de vergüenza de las niñas. Según una investigación realizada en el entorno escolar, el 13% de las niñas han sufrido burlas relacionadas con la menstruación y más del 80% temen ser objeto de burlas, especialmente por parte de sus compañeros varones. Las niñas lo afrontan reduciendo la asistencia a la escuela, la participación y la concentración en el aula durante los periodos^(35)^.

Ante este panorama, es fundamental incluir la perspectiva masculina en las cuestiones relacionadas con la menstruación para promover cambios efectivos. La educación sobre el ciclo menstrual debe comenzar en la escuela, dirigiéndose tanto a los niños como a las niñas. Las personas que no menstrúan expresan interés en aprender sobre el tema^(36)^, y incluir esta cuestión como parte de la educación sexual y las habilidades para la vida no debe ser solo una formalidad, sino una acción concreta. Al educar a los niños sobre la gestión menstrual, crecerán más informados y capacitados para contribuir a la reducción del estigma menstrual, creando entornos escolares más inclusivos y saludables para todos.

Las principales fuentes de información sobre la menarquia para las adolescentes son, en su mayoría, sus madres (39,7%), seguidas de sus amigas (26,6%) y sus maestras (21,8%)^(26)^. Las actitudes y creencias que las familias tienen con respecto a la menstruación influyen significativamente en la forma en que las niñas y los niños comprenden y manejan el ciclo menstrual. Un estudio reveló que la probabilidad de una buena práctica de gestión de la higiene menstrual era mayor entre aquellas que tenían conocimientos sobre higiene menstrual antes de la menarquia y habían hablado sobre el ciclo menstrual con sus padres^(36)^.

Sin embargo, muchas niñas informaron que sus familias, especialmente sus madres o padres, no estaban dispuestas a hablar de estos temas, lo que convertía la menstruación en un tabú dentro del hogar. Esta falta de diálogo franco sobre temas como la variación del flujo menstrual, los artículos para el manejo de la menstruación y los síntomas asociados, tuvo un impacto negativo en la experiencia menstrual de estas niñas, especialmente al comienzo de la adolescencia, cuando aún se están adaptando a la nueva realidad corporal^(36)^.

En este contexto, aunque la dignidad menstrual ha ganado más atención y ha generado políticas públicas, como las mencionadas, los estudios revelan que aún hay lagunas que deben llenarse. Es fundamental considerar la experiencia de otros cuerpos que menstrúan, además de las niñas y mujeres cisgénero^(37)^, así como una perspectiva interseccional del tema, ya que la pobreza menstrual afecta más a las personas que viven en la periferia y a las personas negras^(38)^. Además, existe una necesidad urgente de más investigaciones en Brasil que aborden a los adolescentes en edad escolar, así como que exploren diversas regiones y realidades culturales del país, lo que permitiría una perspectiva más amplia y equitativa sobre el tema. Los estudios futuros también serán esenciales para analizar el impacto del Programa Dignidad Menstrual, que se implementó recientemente, lo que permitirá su mejora.

Como se ha visto en esta investigación, persiste una brecha significativa en la acogida que ofrecen estas políticas de distribución de artículos para el manejo menstrual, especialmente cuando se trata de reconocer y abordar el dolor menstrual que afecta a tantas personas que menstrúan. Esta realidad cotidiana a menudo se subestima y se trivializa, y las dificultades asociadas se naturalizan en lugar de considerarse y abordarse adecuadamente^(39,40)^.

Como limitaciones de este estudio, se puede señalar la falta de un mayor número de respuestas al cuestionario y la consiguiente participación en la ronda de conversación, lo que afectó al poder de inferencia en el análisis cuantitativo. Además, una de las lagunas de la investigación fue no haber involucrado a los adolescentes varones en los grupos focales, lo que podría haber contribuido a una comprensión más amplia e inclusiva de la menstruación, desafiando los estigmas y promoviendo una mayor concienciación entre todos los géneros. Estas limitaciones refuerzan la necesidad de futuros estudios que amplíen el alcance de los participantes y aborden las experiencias de diferentes identidades de género para obtener una visión más completa e inclusiva.

## CONCLUSIÓN

La presente investigación destacó cómo el ciclo menstrual afecta directa e indirectamente la vida cotidiana, escolar y emocional de las adolescentes, generando inseguridad, absentismo escolar y posibles dificultades para acceder a productos para el manejo de la menstruación. Entre los principales resultados, se destaca que más de la mitad de las adolescentes han dejado de ir a la escuela cuando estaban menstruando y han informado sentirse inseguras durante este período. A pesar de que la escuela es una fuente central de aprendizaje sobre el tema, cuestiones como la vergüenza y la estigmatización aún persisten, especialmente por parte de los adolescentes varones. Al relatar las experiencias de las adolescentes, el estudio contribuye a la visibilidad del ciclo menstrual como un tema importante, señalando la necesidad de intervenciones educativas y familiares que amplíen el diálogo y el apoyo, promoviendo una mayor aceptación y comprensión de los retos menstruales. Estos hallazgos refuerzan la necesidad de acciones intersectoriales que integren la educación, la salud y la asistencia social para garantizar la dignidad menstrual, combatir los estigmas y promover entornos escolares más inclusivos y equitativos.

## DISPONIBILIDAD DE DATOS

El conjunto de datos que respaldan las conclusiones de este estudio está disponible previa solicitud al autor correspondiente.
